# Enhanced intensity-based clustering of isomorphous multi-crystal data sets in the presence of subtle variations

**DOI:** 10.1107/S2059798325004589

**Published:** 2025-05-29

**Authors:** Amy J. Thompson, James Beilsten-Edmands, Cicely Tam, Juan Sanchez-Weatherby, James Sandy, Halina Mikolajek, Danny Axford, Sofia Jaho, Michael A. Hough, Graeme Winter

**Affiliations:** a Diamond Light Source Ltd Harwell Science and Innovation Campus DidcotOX11 0DE United Kingdom; b Research Complex at Harwell Harwell Science and Innovation Campus DidcotOX11 0FA United Kingdom; c School of Biosciences University of Birmingham Edgbaston BirminghamB15 2TT United Kingdom; d NE-CAT and Department of Chemistry and Chemical Biology Cornell University Argonne National Laboratory Lemont IL60439 USA; University of Cambridge, United Kingdom

**Keywords:** multi-crystal data sets, data processing, clustering, automation

## Abstract

Multi-crystal processing of X-ray diffraction data has become highly automated to keep pace with the current high-throughput capabilities afforded by beamlines. A significant challenge, however, is the automated clustering of such data based on subtle differences such as ligand binding or conformational shifts. Intensity-based hierarchical clustering has been shown to be a viable method of identifying such subtle structural differences, but the interpretation of the resulting dendrograms is difficult to automate. Using isomorphous crystals of bovine, porcine and human insulin, the existing clustering methods in the multi-crystal processing software *xia*2.*multiplex* were validated and their limits were tested. It was determined that weighting the pairwise correlation coefficient calculations with the intensity uncertainties was required for accurate calculation of the pairwise correlation coefficient matrix (correlation clustering) and dimension optimization was required when expressing this matrix as a set of coordinates representing data sets (cosine-angle clustering). Finally, the introduction of the *OPTICS* spatial density-based clustering algorithm into *DIALS* allowed the automatic output of species-pure clusters of bovine, porcine and human insulin data sets.

## Abstract 2

Bovine, porcine and human insulin data sets are isomorphous in terms of unit cell and symmetry. Through enhancements to the multi-crystal clustering methods in *DIALS*, species-pure data sets are automatically separated by new uncertainty-weighted calculations of pairwise correlation coefficients, algorithm improvements and integration of the *OPTICS* spatial density clustering method.

## 1. Introduction

Multi-crystal X-ray crystallography experiments have seen a resurgence in structural biology to accommodate small, weakly diffracting and/or radiation-sensitive samples (Hirata, 2025 ▸). Simultaneous advances in multi-crystal data processing (Gildea *et al.*, 2022 ▸; Soares *et al.*, 2022 ▸; Assmann *et al.*, 2020 ▸; Yamashita *et al.*, 2018 ▸) have helped to facilitate the related renaissance of room-temperature macromolecular crystallo­graphy, with multi-crystal approaches helping to minimize the effect of radiation damage occurring at lower absorbed doses (Thorne, 2023 ▸; Nave & Garman, 2005 ▸). Room-temperature methods have been demonstrated to be beneficial, allowing structural features to be identified that may be ‘frozen out’ of conventionally collected cryogenic crystals (Helliwell, 2020 ▸; Fraser *et al.*, 2009 ▸; Fischer *et al.*, 2015 ▸). Avoiding the requirements of additional cryoprotectants and manual handling for *in situ* methods also minimizes potential crystal damage (Fischer, 2021 ▸; Mikolajek *et al.*, 2023 ▸) and may be the only tractable approach for certain challenging samples. There is also a growing interest in room-temperature fragment screening to provide experimental data closer to physiological temperature than cryogenic structures for drug development (Skaist Mehlman *et al.*, 2023 ▸; Jacobs *et al.*, 2024 ▸).

While there are a number of advantages to multi-crystal data collection, the recent resurgence in the field can be attributed to faster data-collection times at brighter light sources and developments in processing software which address the associated challenges (Aller *et al.*, 2015 ▸). Establishing a consensus symmetry and consistent indexing mode is nontrivial due to the potential presence of indexing ambiguities and the typically low number of reflections in common between data sets, as well as fewer symmetry-related reflections within each data set. These challenges have been addressed through dimensionality-reduction techniques implemented in numerous software packages (Brehm & Diederichs, 2014 ▸; Diederichs, 2017 ▸; Gildea & Winter, 2018 ▸; Gildea *et al.*, 2022 ▸; Kabsch, 2014 ▸). The removal of outlier data sets and bad images is another challenge that has been well addressed in recent years (Assmann *et al.*, 2016 ▸; Beilsten-Edmands *et al.*, 2020 ▸; Gildea *et al.*, 2022 ▸). One difficulty that remains, however, is the separation of non-isomorphous data sets and the efficient detection of subtle differences between crystallographically isomorphous data sets.

One key technique that has been employed to identify isomorphous subsets within a multi-crystal data collection is hierarchical cluster analysis (HCA). This technique progressively groups together data that are the most alike into clusters, typically visualized using a dendrogram, with the largest cluster containing all data sets. As a result, the vertical axis of a dendrogram is related to how similar the data sets within each cluster are, with the exact nature of this relationship dependent on the statistical descriptors used to characterize the data and the linkage method used to group the data into clusters. Currently available software uses either unit-cell based clustering (such as *BLEND*; Foadi *et al.*, 2013 ▸), intensity-based clustering [such as *ccCluster* (Santoni *et al.*, 2017 ▸), *Cluster*4*x* (Ginn, 2020 ▸) and *XSCALE_ISOCLUSTER* (Assmann *et al.*, 2020 ▸)] or a combination of the two [*KAMO* (Yamashita *et al.*, 2018 ▸; Soares *et al.*, 2022 ▸) and *xia*2.*multiplex* (Gildea *et al.*, 2022 ▸)]. Where a structural model is also available, *Cluster*4*x* has shown that combining information from intensity-based clustering and shifts in C^α^ positions can also result in efficient separation of data (Ginn, 2020 ▸). As for the linkage method, both Ward linkages (Foadi *et al.*, 2013 ▸; Soares *et al.*, 2022 ▸; Matsuura *et al.*, 2023 ▸) and average linkages (Santoni *et al.*, 2017 ▸; Gildea *et al.*, 2022 ▸) have been utilized, although a downside of HCA is that the outcome of the clustering can be very sensitive to the choice of linkage method. Various experiments using these methods have shown them to be effective in obtaining an isomorphous data set within a multi-crystal experiment (Giordano *et al.*, 2012 ▸), although the success of different methods is highly dependent on the type of experiment and the extent of inter-data-set differences.

HCA based on changes in unit-cell dimensions can quickly filter cases of structural non-isomorphism but can miss subtle structural differences such as the presence of a ligand or slight conformational changes (Matsuura *et al.*, 2023 ▸). Therefore, multiple software packages have shifted to using unit-cell clustering as a pre-filtering step prior to performing intensity-based clustering, which is more sensitive to subtle changes (Soares *et al.*, 2022 ▸; Gildea *et al.*, 2022 ▸). Intensity-based clustering is based on pairwise correlation coefficients between all possible data sets. Methods derived from those of Brehm and Diederichs (Brehm & Diederichs, 2014 ▸) have also been implemented in a number of software packages which extend this clustering method to separate random from systematic differences which can cause a change in correlation between data sets (Gildea *et al.*, 2022 ▸; Assmann *et al.*, 2020 ▸). Within the context of *xia*2.*multiplex* (Gildea *et al.*, 2022 ▸), this extension of correlation coefficient intensity-based clustering was given the name ‘cosine-angle clustering’. Where all samples are of high quality, these extended algorithms may not be necessary but should greatly improve the clustering of data sets of varying quality. The power of intensity-based clustering has been demonstrated to separate crystals of apo and ligand-bound protein, crystals of the same protein with different ligands, and crystals with differences in secondary structure (Ginn, 2020 ▸; Soares *et al.*, 2022 ▸; Matsuura *et al.*, 2023 ▸). A significant drawback of this technique, however, lies in the interpretation of the dendrogram, which is often manual and subjective.

Eventually, all data sets must be included in a single cluster, with clusters progressively becoming more internally consistent further down the dendrogram until the data sets are completely separated. Whilst a lower height on the dendrogram implies a higher internal correlation, deciding the exact point at which clusters should be separated to produce meaningful results is not trivial. Typically, two methods for deciding this have been used: having prior knowledge of how many groups there are or deciding a threshold at which to cut the dendrogram (Matsuura *et al.*, 2023 ▸). It is often not known in advance how many different groups lie within the data, so the second method tends to be more useful within a structural biology context. This, however, requires either manual inspection of the dendrogram or a method of determining the cutoff. The former is not ideal given the ever-increasing speed of high-throughput crystallography, and the latter is difficult to generalize. It is also possible that a single threshold may not be appropriate to best separate clusters within a dendrogram; however, an ‘isomorphic threshold’ has recently been proposed within the context of structural biology, where the recommendation has been made to cut the dendrogram at 60–70% of the maximum Ward distance in correlation coefficient HCA (Matsuura *et al.*, 2023 ▸). This threshold is presented as a range, reflecting the fact that the scale of differences investigated will likely vary. They may also be temperature- and sample-dependent, meaning that manual intervention may still be required. Therefore, it would be beneficial to develop an intensity-based clustering method which could identify key clusters without manual inspection, knowledge of the number of groups present, decision on the linkage method or assignation of a threshold.

The cosine-angle clustering method provides a representation of the pairwise correlation matrix in a reduced dimensional space (Gildea & Winter, 2018 ▸; Gildea *et al.*, 2022 ▸). As each data set is represented by a set of coordinates, the entire suite of unsupervised machine-learning clustering methods for spatial data sets becomes available when this intensity-based method is used. Historically, three types of clustering methods have been utilized for spatial data sets: partitioning, hierarchical and density-based (Ester *et al.*, 1996 ▸), although a number of hybrid methods now exist (Ankerst *et al.*, 1999 ▸). Partitioning algorithms attempt to assign all data sets to a cluster, ignoring any potential outliers, and generally require the number of expected clusters as an input parameter. These issues are similar to the disadvantages of hierarchical methods discussed previously. Spatial density-based algorithms, however, have a number of advantages over these other methods. By assuming that clusters are regions of high-density data points, the expected number of clusters does not need to be specified, the clusters can be of any arbitrary shape and outlier data sets can be identified (Ester *et al.*, 1996 ▸). Therefore, this approach was chosen for the determination of significant clusters rather than the typically used HCA methods.

**Figure 1:**
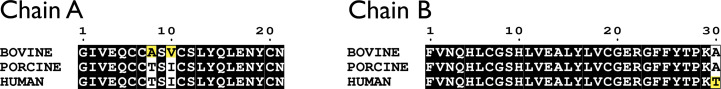
Sequence alignment of bovine, porcine and human insulin for chain A and chain B. Dark shading shows identical amino acids. The less conserved areas are unshaded, with the most different residue highlighted in yellow.

Bovine, porcine and human insulin were selected as test systems to develop these methods for several reasons: insulin crystals grow reproducibly to a high quality, the cubic symmetry provides high multiplicity and completeness for efficient methods development, and different species of insulin form crystals that are crystallographically isomorphous (space group *I*2_1_3 with *a*, *b*, *c* ≈ 78 Å). A sequence alignment of the three species of insulin is given in Fig. 1 ▸. Note that porcine and human insulin are the most similar, only differing by the terminal amino acid in chain B, while the other insulin pair combinations differ by at least two mid-chain amino acids. Studies were conducted using data measured at both room temperature (293 K) and under cryogenic conditions (100 K) to ensure that the methods were widely applicable. In this work, we describe the implementation of new methodologies in the *DIALS* framework for intensity-based isomorphism analysis, which now enable the automated separation of previously inseparable data sets with subtle intensity differences.

## 2. Methods

Evaluations of clustering methodologies were undertaken within the *DIALS* framework, which contains unit-cell clustering algorithms and intensity-based clustering methods from the *dials.cosym* module, which are used as part of the *xia*2.*multiplex* auto-processing pipeline (Winter *et al.*, 2022 ▸; Gildea *et al.*, 2022 ▸). The *cosym* algorithm evaluates systematic and random differences between data sets, either for the purpose of symmetry analysis and indexing ambiguity resolution, or for intensity-based isomorphism analysis of data sets with a consistent symmetry (Diederichs, 2017 ▸; Gildea & Winter, 2018 ▸). Within *xia*2.*multiplex*, the *cosym* algorithm is first used for symmetry assessment and indexing ambiguity resolution. Following scaling of all data sets, the *cosym* clustering is again used for intensity-based isomorphism analysis.

For intensity-based isomorphism analysis, the *cosym* procedure minimizes the function
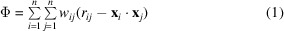
where *r_ij_* is the correlation coefficient between data sets *i* and *j* and the data sets are represented by a set of coordinates **x** in a multidimensional space (where **x**_*i*_ · **x**_*j*_ is the multidimensional inner product), which are optimized from a starting set of random coordinates in the range [0–1] (Gildea & Winter, 2018 ▸). The input to this algorithm is the pairwise matrix of correlation coefficients, and the reduced coordinates from equation (1) are used in *xia*2.*multiplex* to construct a pairwise matrix of cosine angles between data sets for HCA (Gildea *et al.*, 2022 ▸). HCA within *xia*2.*multiplex* has previously used the average linkage method for both the correlation and cosine-angle clustering, although previous reports indicate that the Ward linkage is more suitable as it minimizes chain effects (where clusters grow one data set at a time; Matsuura *et al.*, 2023 ▸; Murtagh & Legendre, 2014 ▸). For all of the examples in this study, both average and Ward linkages were assessed (see supporting information), and the Ward linkage performed significantly better. As a result of these findings, the default linkage in *DIALS* and *xia*2.*multiplex* has been changed to Ward for both the HCA of the *r_ij_* matrix and the pairwise matrix of cosine angles. Note that for HCA a distance metric between data sets must be defined, which we define as 1 − *r_ij_* for correlation clustering and 1 − cos(**x**_*i*_, **x**_*j*_) for cosine-angle clustering. The construction of the pairwise matrix of cosine angles is not required, however, for the proposed analysis in this work using spatial density-based clustering methods. An analogous analysis using equation (1) can also be performed in the *XDS* package using *XSCALE_ISOCLUSTER* (Assmann *et al.*, 2020 ▸). As a development on the previous work, we also introduce the *w_ij_* weights matrix, which can be constant weights or reliability weights, as discussed in the next section.

### 2.1. Weighted calculation of correlation coefficient

When calculating the correlation between two data sets, the correlation coefficient (CC) is defined as the Pearson correlation coefficient between the average (merged) intensities of common reflections between the two data sets (Karplus & Diederichs, 2012 ▸), 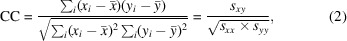
where *x_i_* and *y_i_* are the merged intensities for data sets *x* and *y* for a common symmetry-unique reflection *i* and the sum is performed over common symmetry-unique groups.

In the implementation within *cctbx* (Grosse-Kunstleve *et al.*, 2002 ▸), also used by *DIALS* (Winter *et al.*, 2022 ▸), the merged intensities are calculated as inverse-variance weighted means of the symmetry-equivalent intensities, 
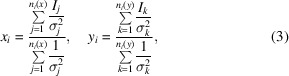
where *I_j_* and σ_*j*_ are the scaled intensity and scaled intensity-uncertainty of an individual observation. While this gives the best estimate of the merged intensity, the uncertainty of this merged intensity is not typically used when calculating merging statistics (such as *R*_p.i.m._) in macromolecular crystallography, which leads to several problems. For a given unique group *i*, the reliability of *x_i_* and *y_i_* will only be approximately the same if the individual uncertainties are similar and *n*_1_ ≃ *n*_2_. For rotation data, where each observation can be modelled and integrated as it passes through the diffraction condition, the uncertainties may indeed be similar; however, for sparser narrow-wedge or still-shot data, the number of common reflections between a pair of data sets in a particular symmetry group become low and there may be a large relative difference between *n*_1_ and *n*_2_. Furthermore, for still-shot (*i.e.* serial) data, the uncertainty in the partiality estimates (which can be a large fraction of the partiality value) results in a further increase in intensity uncertainty, which needs to be accounted for in CC calculations. Even if each *x_i_* and *y_i_* for a symmetry group has the same uncertainty, the effect of ignoring relative uncertainties between different symmetry groups in the CC calculation is profound. Without suitable uncertainty weighting, CC is most sensitive to the merged intensities with high relative uncertainty due to their high variability, when such reflections should in fact be down-weighted by their uncertainty. Therefore, unweighted pairwise correlations become unreliable in the presence of higher uncertainty merged reflections, such as for correlation calculations between small wedges of multi-crystal data or serial data, or for noise-sensitive data-quality indicators such as anomalous correlation coefficients.

When merging a set of symmetry-equivalent observations, the standard error of the weighted mean is given by
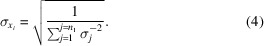
Each term in the Pearson CC can then be weighted as
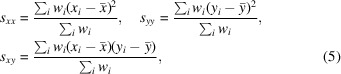

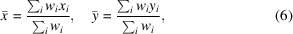
where the weights vector **w** defines the relative weight of each pair of observations,
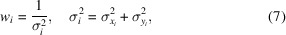
*i.e.* the weight for each symmetry-unique group is the reciprocal of the combined variance of the pair of merged intensities. We note that introducing uncertainty weights into a CC calculation lowers the relative contribution from the higher intensity low-resolution data compared with an unweighted calculation; an approximation for the absolute uncertainty of a merged intensity is 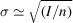, whereas an unweighted CC calculation is equivalent to weighting each merged intensity with equal variance. As such, an unweighted CC calculation is overly sensitive to the strongest intensities as it does not account for the higher absolute uncertainty of stronger reflections. This naturally down-weights the response to subtle differences between data sets. In the rest of this work, we will refer to the inverse-variance weighted CC calculation (equations 4, 5, 6 and 7) with the shorthand ‘σ-weighted’ CC.

Another important consideration for a set of narrow-rotation data sets is that the number of common reflections between a pair of data sets can vary significantly, and therefore it becomes desirable to use reliability weights in the *cosym* objective function (*w_ij_*; equation 1). We use the simple weighting scheme of using the effective sample size *n*_e_ of each pairwise correlation calculation as its relative weight (*i.e.w*_*xy*_ ∝ *n*_e__(*x*,*y*)_, with zero weights for incalculable pairwise correlations). The effective sample size is given by (Kish, 1965 ▸)
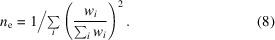
Note that 1 < *n*_e_ ≤ *n*, *i.e.* the effective sample size is lower than the number of common reflections *n* when the symmetry-group weights are unequal and approaches one in the limit of one merged intensity having much lower uncertainty than all other intensities. Pairwise correlation calculations are performed after applying a consistent resolution filter across all data sets, determined as the higher resolution of conservative filters mean(*I*)/mean(σ) > 4.0 and CC > 0.6 calculated on the whole data set.

### 2.2. Automated evaluation of clustering dimensionality

In the initial implementation of intensity-based isomorphism analysis in *dials.cosym*, the target use case was the distinction of the most isomorphous cluster of data sets for a single protein crystal system, and the objective function (equation 1) was minimized in two dimensions. However, methods from Diederichs show that as the number and type of systematic differences present increase, the required number of dimensions also needs to increase (Diederichs, 2017 ▸), and therefore a general solution is required to determine the number of dimensions for intensity-based isomorphism analysis.

The methods presented by Diederichs use the number of significant eigenvalue/eigenvector pairs of the matrix of correlation coefficients as the number of required dimensions (Diederichs, 2017 ▸). The related approach taken in *dials.cosym* for symmetry analysis (Gildea & Winter, 2018 ▸) is to analyse the residual after minimizing equation (1) as a function of the number of dimensions, as it relates to how well the data are described by that number of dimensions: a large value at a given dimension indicates that more dimensions are needed to adequately describe the systematic differences within the data.

For intensity-based isomorphism analysis in an unknown number of dimensions, we adopt the same approach: equation (1) is minimized for all possible dimensions for the data set (1 through to the number of data sets) and the trend of the residuals is analysed. In practice, the maximum number to be tested is limited to 50 for computational efficiency. The optimal number of dimensions to perform the analysis is then defined as the elbow point of this curve, representing the point where all significant residuals have been accounted for. The algorithm for determining the elbow point of a curve is the same approach as that used to determine the per-image resolution cutoff in *DISTL Spotfinder* (Zhang *et al.*, 2006 ▸) and also implemented in *DIALS*. Once the optimal number of dimensions has been determined, the *cosym* coordinate solution from this number of dimensions can be used for hierarchical clustering analysis based on cosine-angle or spatial density-based clustering analysis, as described in the next section.

### 2.3. Identification of significant clusters using the *OPTICS* algorithm

**Figure 2:**
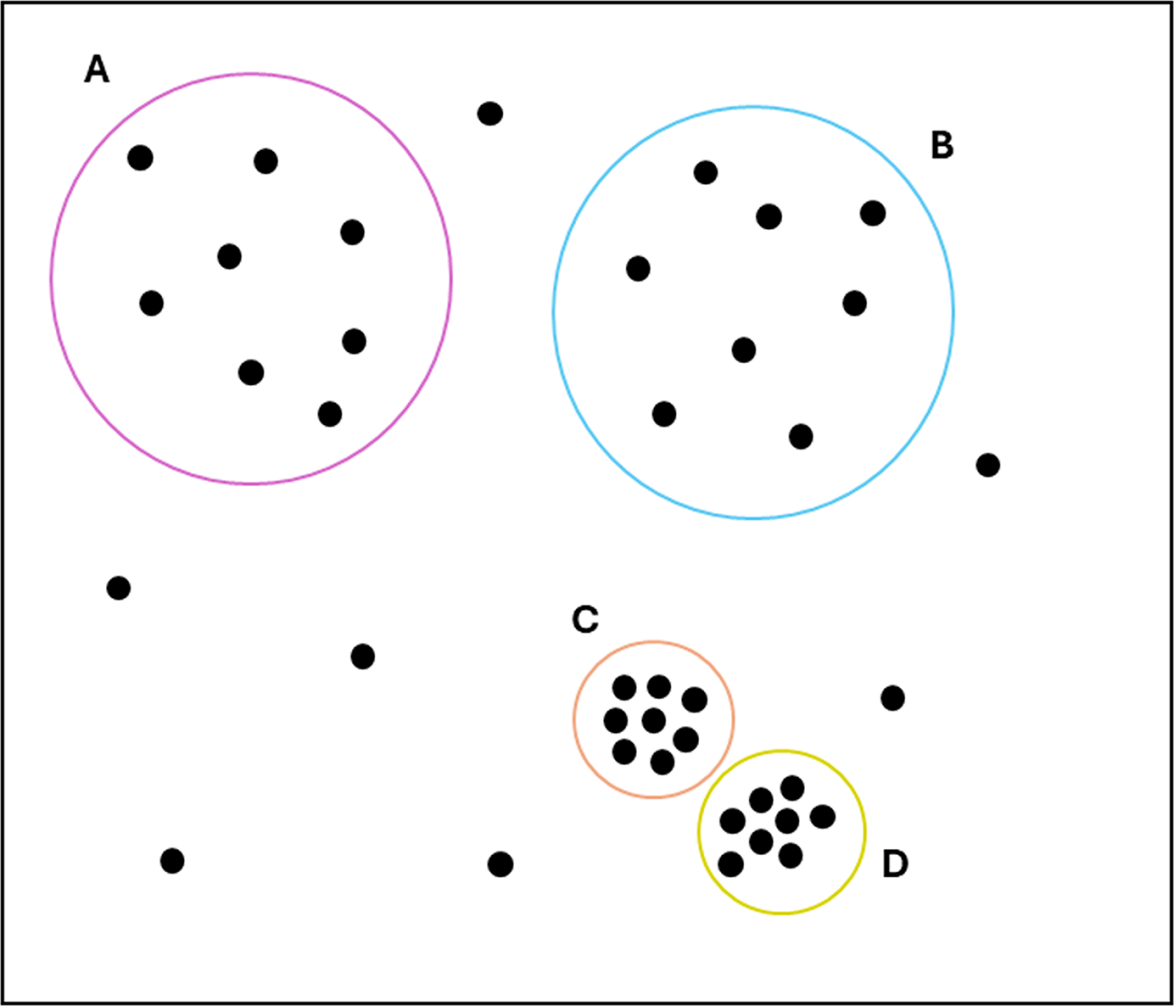
Graphical representation of the spatial density-based clustering results produced by *OPTICS* (Ankerst *et al.*, 1999 ▸). Data points are represented as black dots, with circles of different colours (A, B, C and D) representing clusters. By not including a global density requirement, clusters of different spatial densities can be automatically separated (A, B, C and D), whereas a global density requirement would either join C and D together or omit A and B entirely. The same concept applies in higher dimensions.

Within the family of spatial density-based clustering algorithms, several different methods exist. Perhaps the most well known is the *DBSCAN* algorithm (Ester *et al.*, 1996 ▸), which has all of the aforementioned advantages of spatial density-based clustering algorithms. It does have a significant drawback, however, in that it applies a global density parameter, and thus assumes that all clusters have the same underlying spatial density, which is unlikely to hold true for all cases. There are two commonly used algorithms that improve the base principles of *DBSCAN* to account for clusters of different spatial density and thus reduce the requirement for input parameters: *HDBSCAN* (Campello *et al.*, 2013 ▸) and *OPTICS* (Ankerst *et al.*, 1999 ▸). The algorithms have similarities, although *HDBSCAN* may be more computationally expensive, and the *OPTICS* algorithm has other distinct advantages which suit this case better (Schubert & Gertz, 2018 ▸). *OPTICS* is an unsupervised machine-learning algorithm and does not produce a list of clusters per se. Instead, it produces an augmented ordering of points representing the structure of the data according to its spatial density (Ankerst *et al.*, 1999 ▸). This removes the need for a global density parameter to be defined, meaning that clusters of different spatial density can be automatically identified (a graphical representation is shown in Fig. 2 ▸). A ‘reachability plot’ is also produced which is used for cluster identification. In these plots, data sets that belong to a cluster have low reachability distances and thus appear as valleys, and boundaries between clusters have high reachability distances and thus appear as spikes. Interpretation of these plots can be automated by quantifying the slope between consecutive ordered data sets (ξ) alongside criteria such that when a set of conditions are met the cluster boundary is automatically defined (Ankerst *et al.*, 1999 ▸; Schubert & Gertz, 2018 ▸).

In *DIALS*, clustering with *OPTICS* is achieved by using the implementation (https://scikit-learn.org/dev/modules/generated/sklearn.cluster.OPTICS.html) from the scikit-learn Python package (Pedregosa *et al.*, 2011 ▸), which classifies each data set as either belonging to a cluster or as being an outlier point; the identified clusters are then separated within *DIALS* to allow further scaling and merging of individual clusters. The input data for *OPTICS* clustering are the optimized *N*-dimensional *cosym* coordinates, while we adjust the *s*_min_ parameter, which affects which data points are considered to be core points in the *OPTICS* algorithm, based on the heuristic
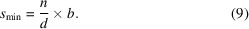
Given that the number of systematic differences present between the data sets (*n*) is roughly estimated by the number of dimensions optimized through the *cosym* minimization procedure (*d*), the value for the minimum number of data sets for a cluster (*s*_min_) can be estimated using a tailorable parameter (*b*) with a default value of 0.5. Changing this buffer parameter is only likely to be needed when the groups present in the data have very different populations (a value of 0.5 was used for all examples in this work), and a floor value of 5 is used for the minimum number of data sets. If *b* is set to be too large, then this assumes that the number of dimensions is a perfect proxy for the number of groups, and that all groups are of equal size. Conversely, if *b* is too small then there is the risk that too many clusters may be identified. The *s*_min_ parameter ultimately controls how rugged the ‘reachability plot’ is. This is, however, also intertwined with the ξ parameter (default ξ = 0.05), which controls the gradient of the points a cluster starts and ends at on the ‘reachability plot’. In practice, larger ξ values will generally be less sensitive to features and be restricted to top-level clusters, whereas lower ξ values will increase the sensitivity to spikes in the reachability plot and find more clusters (the default of 0.05 was used for all examples in this work). Both of these parameters (*b* and ξ) are accessible for users of the software to alter through the command-line interface, and visual examples of the effects of altering these parameters on the *OPTICS* reachability plots are provided in the supporting information.

## 3. Results and discussion

### 3.1. Separation of bovine and human insulin

**Figure 3:**
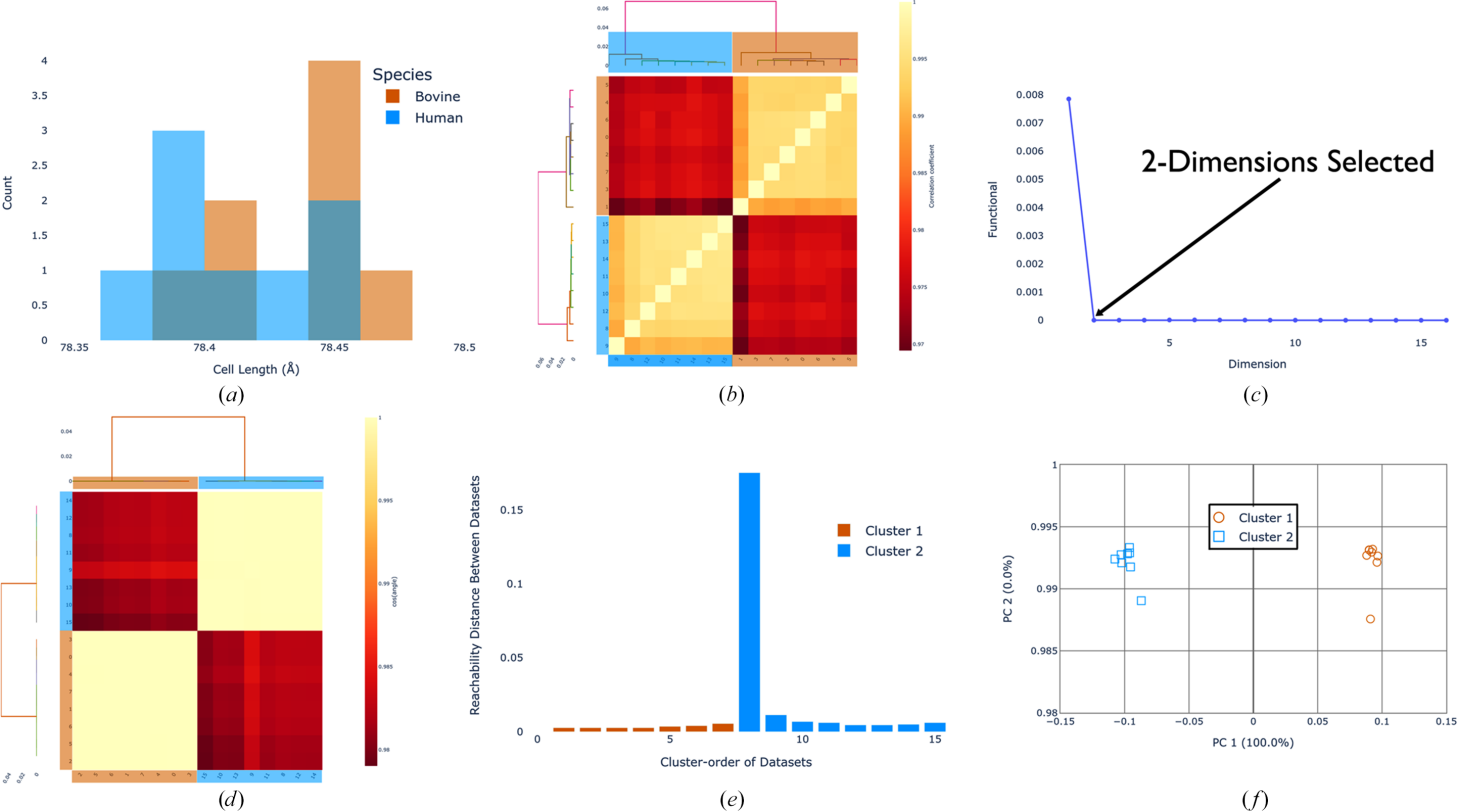
(*a*) Unit-cell dimensions plotted as a histogram to demonstrate overlap between species. (*b*) Correlation-based HCA using the σ-weighted CC algorithm. (*c*) Objective function residual (equation 1) for each tested dimensionality. The automatically selected dimension is highlighted, which is the first dimension where the residual drops into the noise level as determined by the algorithm described in Section 2.2. (*d*) Cosine-angle HCA analysed in two dimensions using the σ-weighted CC algorithm. (*e*) *OPTICS* reachability plot for data sets ordered by the cluster that they belong to. A large spike in the reachability distance corresponds to a cluster boundary. (*f*) Two-dimensional plot of the optimized *cosym* coordinates with the identified clusters colour-coded. The coordinates have been rotated to align the axes with the eigenvectors derived from principal component analysis. Data sets corresponding to bovine insulin are orange and data sets corresponding to human insulin are blue. Dendrogram links have colours that are randomly allocated and are not representative of groups.

To evaluate the performance of the new clustering algorithms implemented through *xia*2.*multiplex*, eight high-quality data sets each of bovine and human insulin (the most diverse pair in this study) were measured at room temperature (293 K) on the VMXi beamline at Diamond Light Source (Sanchez-Weatherby *et al.*, 2019 ▸; Mikolajek *et al.*, 2023 ▸; Sandy *et al.*, 2024 ▸). Diffraction data from 20° wedges were measured and the data were processed using the automatic pipelines. Analysis was performed using these clustering algorithms (see the supporting information for statistics and data-collection parameters). Significant overlap of unit-cell lengths was observed between the two species of insulin (Fig. 3 ▸*a*), and subsequent unit-cell clustering was unable to separate these species (see the supporting information). The merging statistics including all data sets also did not indicate any obvious issues related to non-isomorphism based on data-quality indicators (see the supporting information). The correlation- and cosine-angle-based HCA, however, clearly identified the two groups present (Figs. 3 ▸*b* and 3 ▸*d*), supporting the assertion that the subtle difference between isomorphous bovine and human insulin can be detected using this method. Here, the heatmap is a direct representation of the pairwise correlation matrix (for correlation clustering) or the pairwise cosine-angle matrix (for cosine-angle clustering), with the resulting HCA dendrograms shown on the top and left-hand side for reference. As it was found to enhance the difference between groups, σ-weighting (as in Section 2.1) was applied, although it did not change the conclusions (a comparison is provided in the supporting information). The cosine-angle clustering was performed on two-dimensional coordinates as automatically identified by the algorithms described in Section 2.2. The residual from the minimization of the objective function (equation 1) is plotted for each tested dimension (Fig. 3 ▸*c*), clearly showing that some significant residual remains when only one dimension is used, but for two or more dimensions all fluctuations are within the level of noise. Analysis with the embedded *OPTICS* algorithm identified two clusters with correctly separated insulin species with no outliers (Fig. 3 ▸*f*). Here, the coordinates from the *cosym* analysis have been rotated to align the axes with the eigenvectors identified using principal component analysis. The percentage of variation explained by each principal component is listed, and this is now the default output in *DIALS* for intensity-based clustering analysis. The reachability plots also identified a clear boundary between two tightly packed clusters, as indicated by a spike in the plot (Fig. 3 ▸*e*). The cluster boundary is included in cluster 2 due to the *OPTICS* ordering and definition of the reachability. A high reachability distance means that the data set is far away from the preceding data set. The fact that the second blue data set has a very low reachability distance means that it must be close to the first blue data set by nature of how the algorithm orders data sets.

### 3.2. Separation of bovine, porcine and human insulin

#### 3.2.1. Cryogenic data

**Figure 4:**
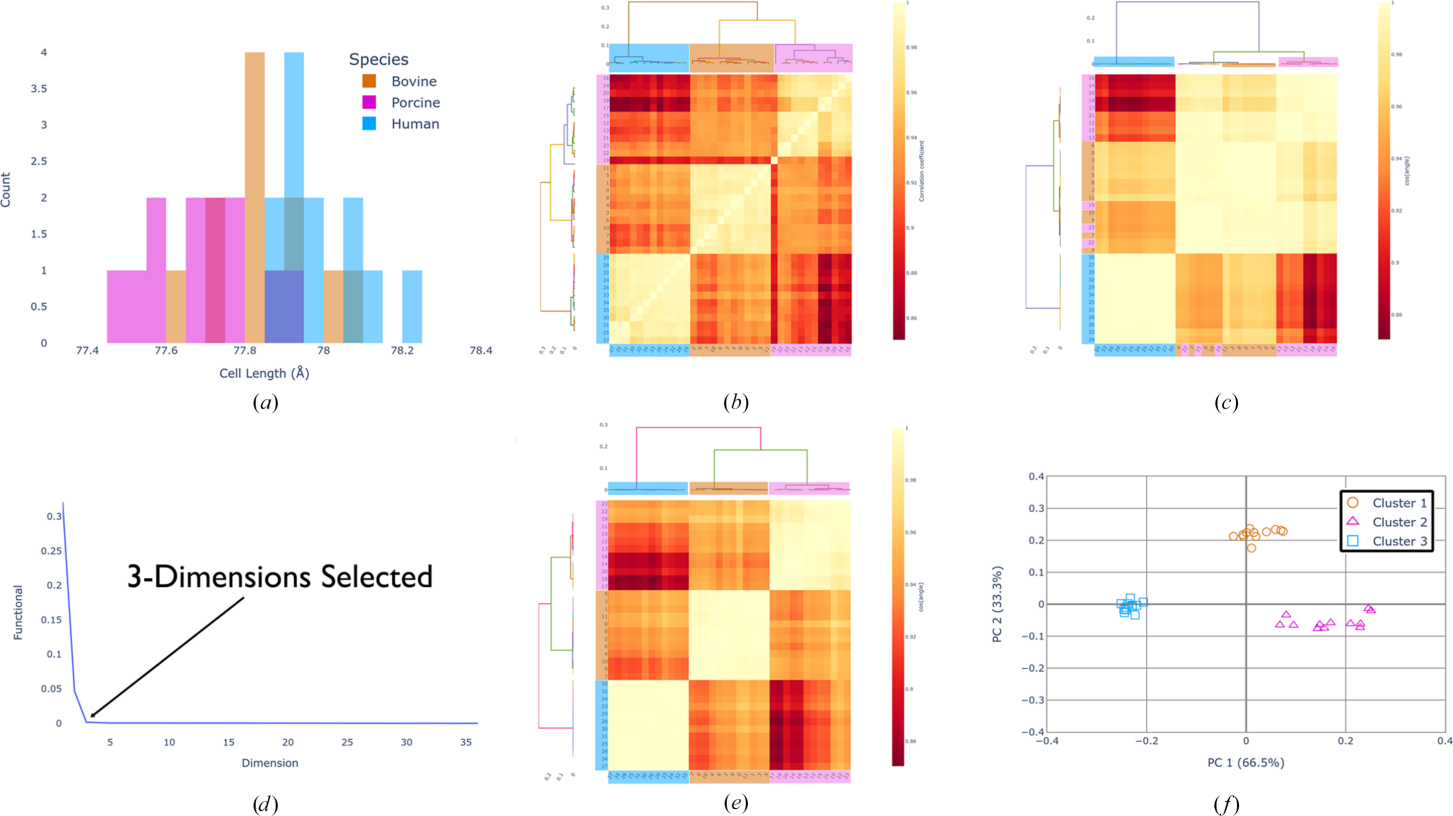
(*a*) Unit-cell dimensions plotted as a histogram to demonstrate overlap between species. (*b*) Correlation-based HCA using the σ-weighted CC algorithm. (*c*) Cosine-angle HCA analysed in two dimensions using the σ-weighted CC algorithm. (*d*) Objective function residual (equation 1) for each tested dimensionality. The automatically selected dimension is highlighted, which is the first dimension where the residual drops into the noise level as determined by the algorithm described in Section 2.2. (*e*) Cosine-angle HCA analysed in three dimensions using the σ-weighted CC algorithm. (*f*) The optimized multi-dimensional *cosym* coordinates, projected in two dimensions, with the analysis performed in three dimensions. The coordinates have been rotated to align the axes with the two most significant eigenvectors derived from principal component analysis. Data sets are coloured according to clusters identified using the *OPTICS* algorithm [bovine (orange), porcine (pink) and human (blue) insulin]. Dendrogram links have colours that are randomly allocated and are not representative of groups. For the other 2D projections of the coordinate plot, see the supporting information.

To further assess the ability of these methods, 12 crystals each of bovine, porcine and human insulin were measured. The addition of porcine insulin presents a greater challenge as it only differs by one terminal amino acid when compared with human insulin. This experiment was performed at cryogenic temperature (100 K), in part to minimize flexibility at the end of chain B, a key point of difference between the samples. Diffraction data (10° wedges) were measured on the I24 beamline at Diamond Light Source and processed together (see the supporting information for data-collection and processing details). Once again, plotting the unit-cell lengths for all three insulin species as a histogram demonstrates clear overlap (Fig. 4 ▸*a*), consistent with the fact that unit-cell clustering could not cleanly separate these species (see the supporting information). The correlation-based HCA, however, was able to clearly distinguish all three species of insulin (Fig. 4 ▸*b*), while the cosine-angle clustering provided an interesting result when performed with the former standard *xia*2.*multiplex* setting of two-dimensional cluster analysis. While the human insulin data sets formed a well separated cluster (Fig. 4 ▸*c*), the bovine and porcine data sets were mixed, likely due to the existence of multiple systematic differences that could not be adequately described in two dimensions. When the clustering is performed with the auto-selected three dimensions (Fig. 4 ▸*d*), the bovine and porcine data sets separate correctly (Fig. 4 ▸*e*). This clearly demonstrates the need for flexible dimensionality when performing intensity-based clustering. While in this case both the correlation and cosine-angle clustering gave the same result, the three groups are more tightly defined in the cosine-angle clustering. The *OPTICS* algorithm identified three clusters with no outliers (Fig. 4 ▸*f*), with all species of insulin correctly separated. As in the previous example, σ-weighting was used, although not strictly required, as it enhanced differences between groups (comparisons are provided in the supporting information).

#### 3.2.2. Room-temperature data

**Figure 5:**
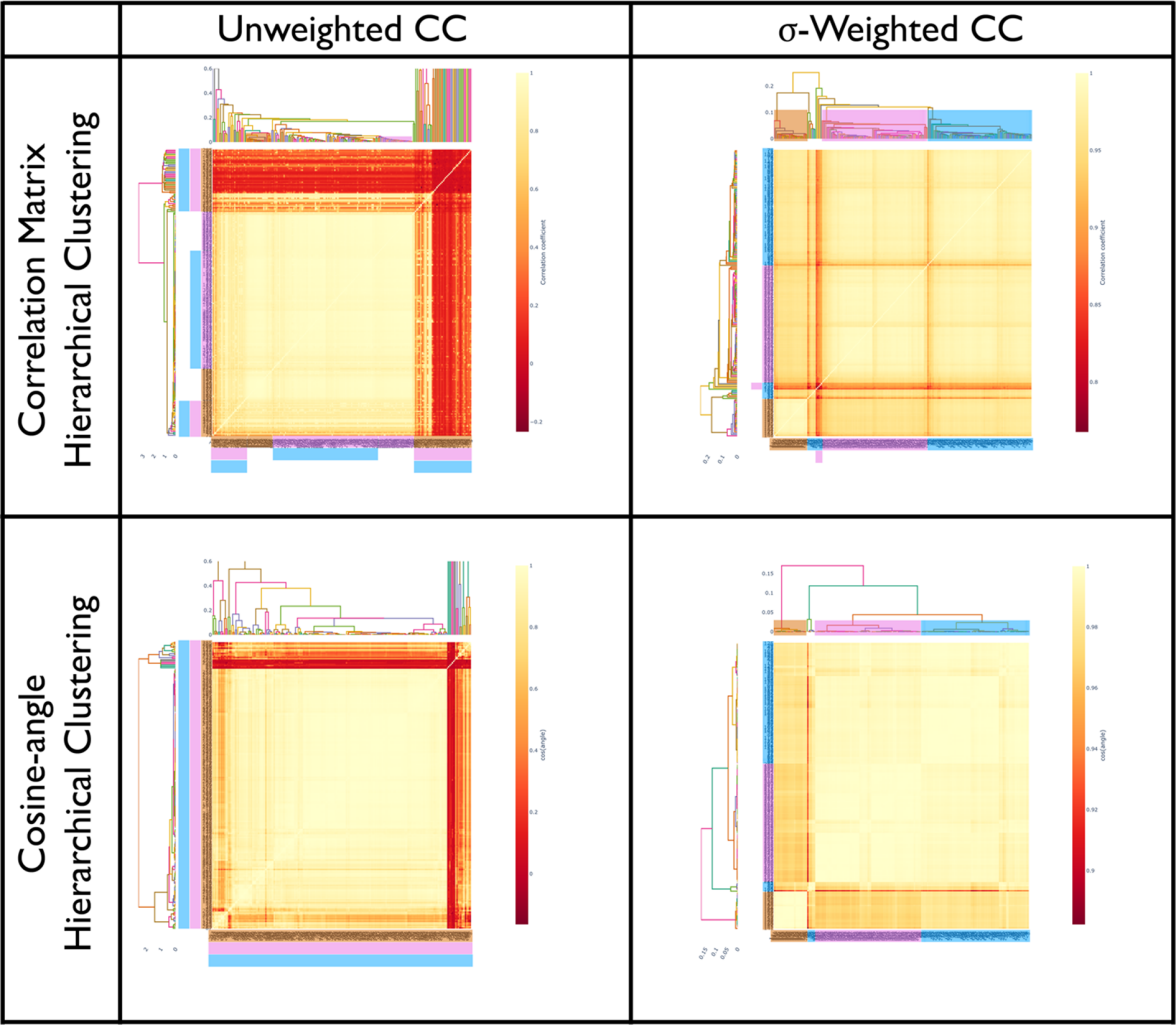
Comparison of HCA analysis of bovine, porcine and human insulin using *xia*2.*multiplex* with and without CC σ-weighting as described in Section 2.1. Data sets corresponding to bovine insulin are highlighted in orange, data sets corresponding to porcine insulin are highlighted in pink and data sets corresponding to human insulin are highlighted in blue. Dendrogram links have colours that are randomly allocated and not representative of groups. The top dendrograms for the unweighted heatmaps show only the lower part of the dendrogram to improve visualization, whereas the full dendrograms are shown to the left.

To evaluate the new methodologies on a typical high-throughput experiment, *i.e.* room-temperature (293 K) *in situ* data collection on the VMXi beamline, data sets of 60° rotation wedges were collected from a large number of bovine, porcine and human insulin crystals and processed together using the same methodology. Uneven numbers of each type of crystal were measured to increase the complexity of the clustering for testing purposes. When the clustering was initially performed in *xia*2.*multiplex*, well-separated clusters for the three different species could not be distinguished without applying σ-weighting to the CC calculations (Fig. 5 ▸). This demonstrates the need for appropriate weighting procedures when multi-crystal data sets increase in size with a greater variation in quality, and when differences may be very subtle, as is the case where porcine and human insulin only differ by a terminal amino acid. This example also demonstrates some of the ambiguity associated with HCA. In both the correlation and cosine-angle clustering, there are clear substructures within the porcine and human insulin groups, so the best place to cut the dendrogram is not clear without prior knowledge or additional analysis.

**Figure 6:**
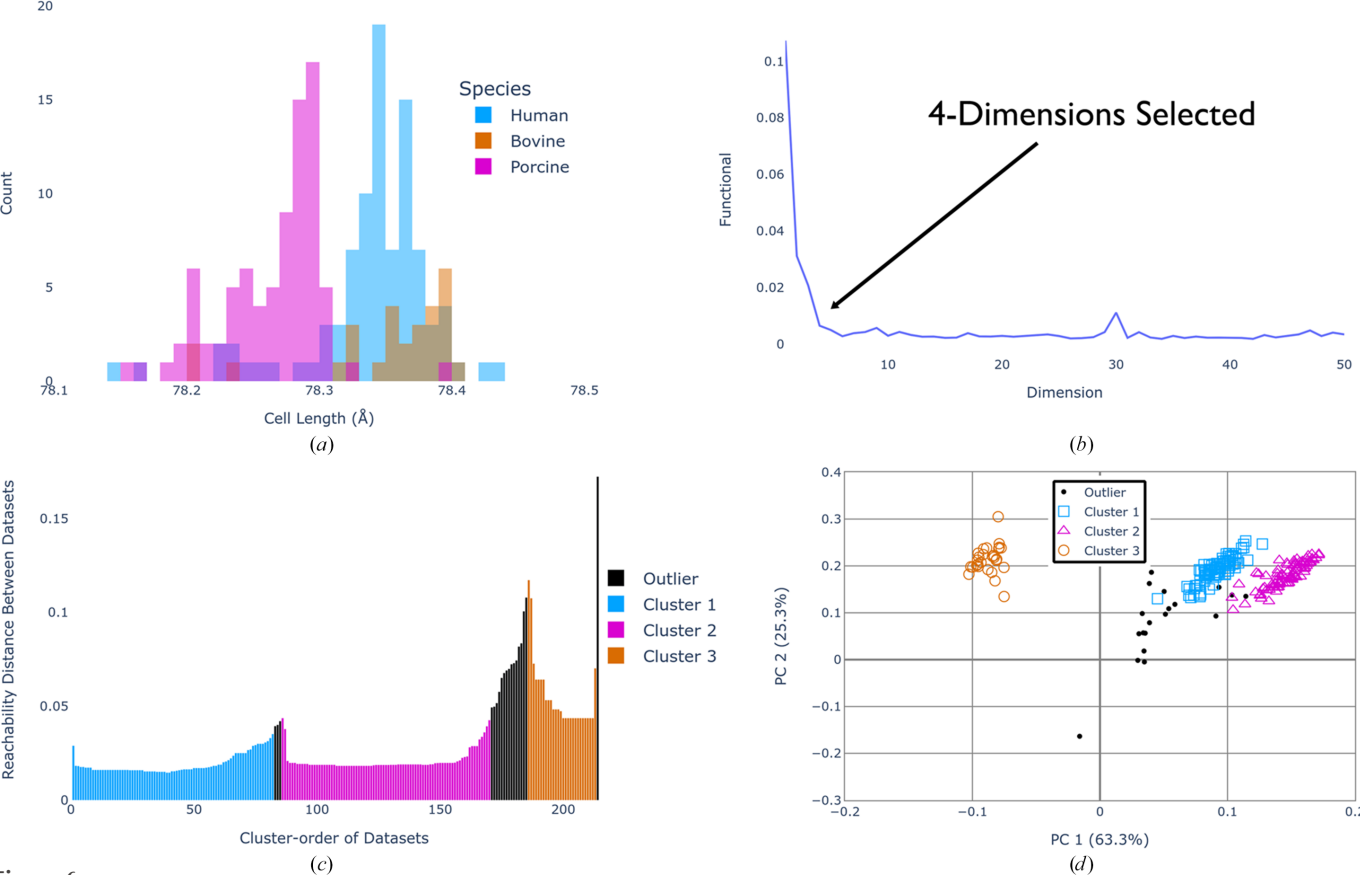
(*a*) Unit-cell dimensions plotted as a histogram to demonstrate overlap between species (some outliers beyond the plotted *x*-axis range were removed for visual clarity). (*b*) Objective function residual (equation 1) for each tested dimensionality. The automatically selected dimension is highlighted, which is the first dimension where the residual drops into the noise level as determined by the algorithm described in Section 2.2. (*c*) *OPTICS* reachability plot for data sets ordered by the cluster that they belong to. A large spike in the reachability distance corresponds to a cluster boundary. (*d*) The optimized multi-dimensional *cosym* coordinates, projected in two dimensions, with the analysis performed in four dimensions. The coordinates have been rotated to align the axes with the two most significant eigenvectors derived from principal component analysis. Data sets are coloured according to clusters identified using the *OPTICS* algorithm [bovine (orange), porcine (pink) and human (blue) insulin]. For the other 2D projections of the coordinate plot, see the supporting information.

As with the previous examples, comparison of the unit-cell lengths for the known species results in significant overlap (Fig. 6 ▸*a*), corresponding to the inability of unit-cell clustering to clearly separate the species (see the supporting information). When the dimensions were automatically optimized for this data set, it was found that four dimensions were needed to account for the main features of the *r_ij_* matrix (Fig. 6 ▸*b*). In the corresponding heatmap (Fig. 5 ▸), it is clear that species-pure groups are observed, as well as some outlier groups. The *OPTICS* analysis identified three species-pure groups (populations of 83, 85 and 28) as well as 19 outlier data sets (Figs. 6 ▸*c* and 6 ▸*d*). This result demonstrates the power of this spatial density-based clustering method: one group was significantly smaller and less dense, but *OPTICS* can account for variations in cluster size and density. The reachability plot produced from the *OPTICS* analysis also highlights some other advantages over alternative spatial density and HCA methods. Under visual inspection, the cluster boundaries are straightforward to identify and the presence of multiple data sets with large reachability values at these boundaries is indicative of noise (as in Fig. 6 ▸*c*). Thus, there is a clear region of data sets which could be outliers, whereas this can be more open to interpretation in a dendrogram representation. By modifying the ξ and *b* parameters, the user has some control over how clusters are defined, with the reachability plots providing guidance (although the default ξ value of 0.05 and *b* value of 0.5 should be appropriate in most cases; Ankerst *et al.*, 1999 ▸; Schubert & Gertz, 2018 ▸). It is worth noting that such clear visualization of the clusters with clear tailoring of parameters is not available through *HDBSCAN*, which is why, despite the two being known for producing similar results, *OPTICS* was chosen.

### 3.3. Quantification of the methodology

#### 3.3.1. Scale of the observed differences

In this study, isomorphous crystals from three species of insulin could be automatically separated where only 1–3 amino acids are different. In terms of molecular weight, this represents a difference of 74 Da when comparing bovine and human insulin, 44 Da when comparing bovine and porcine insulin and only 30 Da when comparing porcine and human insulin. These masses represent 1.3%, 0.8% and 0.5% of the insulin proteins, respectively (although these do not represent lower limits of detection as clustering was successful in all cases). A clear application of this work is the automatic separation of apo versus ligand-bound crystals. Typical fragment-based drug-design campaigns soak in compounds with weights of <300 Da (Bon *et al.*, 2022 ▸); therefore, it is highly feasible that such compounds could be detected using this method, depending on the size of the protein.

#### 3.3.2. Comparison of clustering methods

Quantitative comparison of the different clustering methods presented was undertaken using standard measures: both the Davies–Bouldin score (https://scikit-learn.org/stable/modules/generated/sklearn.metrics.davies_bouldin_score.html; Davies & Bouldin, 1979 ▸) and V-Measure (https://scikit-learn.org/stable/modules/generated/sklearn.metrics.homogeneity_completeness_v_measure.html; Rosenberg & Hirschberg, 2007 ▸) methods were used via the scikit-learn Python package (Pedregosa *et al.*, 2011 ▸). The Davies–Bouldin score provides a measure of similarity of clusters, where lower scores correspond to denser groups with better separation. The score is calculated based on the data (*r_ij_* for correlation clustering and the optimized *cosym* coordinates for both the cosine angle clustering and *OPTICS* methods) as well as the labels assigned by each method (HCA or *OPTICS*). While useful for characterizing clusters, this method does not have any knowledge of what the ‘correct’ labels for each cluster are; therefore, V-Measure analysis was also undertaken, which compares the clustering outcome to known labels. Possible values range from 0 to 1, where 1 implies perfect agreement with a known classification.

A requirement for both of these statistical methods is generating labels for each data set based on the clustering method. While this occurs automatically for the *OPTICS* clustering, the current *DIALS* implementation does not assign a threshold for HCA in order for data sets to be assigned to clusters. As previously discussed, an ‘isomorphic threshold’ has been proposed, suggesting that correlation dendrograms should be cut at 60–70% of the maximum Ward distance (Matsuura *et al.*, 2023 ▸). Therefore, this analysis was undertaken for each data set at both 60% and 70% of the maximum height in both the correlation and cosine dendrograms. While the original proposal for the ‘isomorphic threshold’ was only optimized for correlation-coefficient HCA, the same cutoff values have been applied to the cosine-angle HCA for comparison. For the room-temperature comparison of bovine and human insulin (Section 3.1) and the cryogenic comparison of bovine, porcine and human insulin (Section 3.2.1), correct clustering occurred within this threshold. This was not the case for the comparison of room-temperature bovine, porcine and human insulin data sets (Section 3.2.2); therefore, appropriate thresholds were manually identified for the purpose of comparison between methods. Davies–Bouldin scores and V-Measures were calculated for all identified thresholds for all data sets and clustering methods (Table 1 ▸).

One noticeable trend is that the Davies–Bouldin scores for the cosine-angle HCA are far lower than the corresponding correlation HCA when the V-Measures are equivalent (*i.e.* when the ‘correctness’ is equivalent between the two methods). This follows the visual trends in the dendrograms that the cosine-angle clusters are denser and better separated compared with the correlation dendrograms. Clearly, the separation of random and systematic error via the *cosym* method aids in cluster partition. The next conclusion to draw using the V-Measures is that the proposed 60–70% threshold does not hold for all data sets in this study. While the proposed threshold could be applied to subclusters, as performed previously (Matsuura *et al.*, 2023 ▸), continuing to apply the cutoff further down the dendrogram is not efficient for automation and thus not explicitly tested. The two smaller data sets (Sections 3.1 and 3.2.1) are expected to give perfect V-Measures, and any deviation from 1 in this case is a failure of the clustering method, while some deviation is expected for the large room-temperature data set (Section 3.2.2) due to the additional uncertainty in the data and the expected presence of outliers. The large room-temperature data set (Section 3.2.2) only provides reasonable V-Measures in the correlation HCA at 47% of the maximum Ward distance. Furthermore, this study verifies that the isomorphic threshold does not apply to cosine-angle clustering, as reasonable V-Measures are achieved where a threshold of 18% of the maximum Ward distance.

As for the *OPTICS* method, the scores match the cosine method when perfect clustering is achieved (V-Measure = 1). For the large room-temperature data set (Section 3.2.2), the *OPTICS* scores are comparable to using a manually defined cutoff in either correlation or cosine-angle HCA, as there is likely to be some range of ‘true’ values of the V-Measure depending on how one chooses to classify outliers. However, these scores were achieved automatically using *OPTICS*, whereas manual intervention was required to achieve similar quality using HCA, with previously defined automation guidelines failing for this data set. Therefore, while both HCA and *OPTICS* have the capability to perform similarly for these data sets, the fact that only *OPTICS* was able to achieve this without human intervention highlights the advantages of this method over traditional HCA. It is also worth noting that the exact value of the Davies–Bouldin score cannot be used to automate HCA, as for all cases with high V-Measures the values range from 0.04 to 1.492. This scoring system is best limited to method comparison, further highlighting the challenges in automating HCA and the advantage of using a method such as *OPTICS*.

#### 3.3.3. Common reflection requirement

All insulin species in this study are from crystals of high symmetry, with many common reflections present in small wedges of data. To investigate the applicability of this technique to lower symmetry systems, where less common reflections are present in small wedges, the number of common reflections required for successful clustering of this data was quantified. For the separation of bovine and human insulin (Section 3.1), the number of common unique reflections between pairs of data sets varied from 4723 to 6209, with a mean of 5479, using an automatic resolution filter of 1.72 Å. When taking uncertainties into account, the effective number of unique observations *n*_e_ varied from 690 to 1644, with a mean of 1112. The impact of the number of common reflections required was investigated by successively cutting back the integrated data to the first *n*° of data. It was found that successful clustering via *OPTICS* could still be achieved down to 1.2° of rotation, where the number of common unique reflections between pairs of data sets varied in the range 48–282 (mean 104), with an automatic resolution filter of 1.89 Å and effective sample sizes *n*_e_ in the range 87–133 (mean 40). Below this, the dimensionality assessment selects a higher number of dimensions and *OPTICS* clustering does not identify the correct clusters.

For the separation of bovine, porcine and human insulin data sets at 100 K (Section 3.2.1), the number of common unique reflections between pairs of data sets varied from 6497 to 12 102, with a mean of 9306, using an automatic resolution filter of 1.26 Å. *n*_e_ varied from 1174 to 5481, with a mean of 3188. Again, the effect of reducing the number of common reflections was investigated by successively cutting back the integrated data to the first *n*° of data. In this case, successful *OPTICS* clustering was still achieved down to 0.8° of rotation, where the number of common unique reflections between pairs of data sets varied in the range 53–244 (mean 86), with an automatic resolution filter of 1.48 Å and effective sample sizes *n*_e_ in the range 4–90 (mean 40). Below this, some data sets start to become classed as outliers.

For the separation of bovine, porcine and human insulin data sets at 293 K (Section 3.2.2), the number of common unique reflections between pairs of data sets varied from 4173 to 8634, with a mean of 7651, using an automatic resolution filter of 1.63 Å. *n*_e_ varied from 356 to 4308, with a mean of 2432. This data set exhibits much higher internal variation, as shown by the presence of clustering outliers; therefore, when the rotation range of the integrated data is cut back, it was found that comparably successful clustering could only be achieved after additional scaling and filtering, with some previously classified data sets now being classed as outliers. For example, processing only the first 20° of data resulted in *OPTICS* classification into three clusters (populations 52, 19 and 28) and 116 outliers. With scaling and filtering turned on (*xia*2.*multiplex* options filtering.method=deltacchalf stdcutoff=3.0), 19 data sets were removed and subsequent clustering analysis found three species-pure clusters (populations 76, 80 and 27) and 13 outlier data sets. We note that the higher variability in this room-temperature data set, compared with the first example data set, is due to variability in the centring accuracy of the data collections. As the rotation range is ±30°, there is a higher chance that a crystal rotates out of the beam due to uncertainty in the sample position determined by automated optical centring in *in situ* plates. The effect of centring variability was seen in the initial *xia*2 processing, where some sweeps were automatically cut to a reduced scan range due to blank images, and in scaling, where the overall scale factors for some crystals varied by an order of magnitude or reduced to zero, indicating that some crystals passed into or out of the beam during the rotation range or had large changes in their diffracting volume. Despite these effects, all integrated data sets were used as input to *xia*2.*multiplex* as a realistic test of routine *in situ* data collection and processing.

These examples therefore demonstrate that for high-quality diffraction data the clustering methodologies presented are a highly sensitive technique, where small structural differences can be detected with a small amount of data. The third example, which is more representative of routine room-temperature *in situ* experiments, demonstrates that small structural changes can still be detected, but outlier handling via further scaling and filtering are important to discover clusters as the inherent noise level increases.

#### 3.3.4. Calculation costs

The computational cost of different aspects of the analyses was also investigated. The most computationally expensive part is the calculation of the *r_ij_* matrix, which is required for all clustering methods evaluated in this study (correlation HCA, cosine HCA and *OPTICS*). This process scales as *n*^2^, where *n* is the number of data sets. The dimension optimization is the next most time-intensive calculation, although this could be greatly minimized if needed by specifying the number of dimensions, as a single minimization is orders of magnitude faster than the calculation of the *r_ij_* matrix. The *OPTICS* analysis scales linearly and is slower than HCA by 2–3 orders of magnitude depending on data-set size. HCA analysis is very fast, and does not have a clear scaling behaviour with number of data sets. While this is a computational advantage, this does not currently include the capacity for robust automatic clustering, and the computational trade-off for using *OPTICS* is insignificant when considering that it is still far faster than the calculation of the *r_ij_* matrix (see the supporting information for time-profiling results). Clearly there is scope for improving the computational efficiency of this approach, but with regard to the different clustering methods (correlation HCA, cosine HCA and *OPTICS*), the requirements are comparable.

## 4. Implementation and availability

The algorithms presented here are currently available within the standalone *DIALS* program *dials.correlation_matrix*, as well as within the *xia*2.*multiplex* multi-crystal data-reduction and analysis pipeline in *DIALS* versions 3.24.0 and above (https://dials.github.io/installation.html). σ-weighted calculation of pairwise CC values and dimension optimization are now used by default within both software packages. To output identified clusters as *DIALS* experiment and reflection files in *dials.correlation_matrix*, set the input significant_clusters.output=true. To output scaled clusters in *xia*2.*multiplex*, set the input clustering.output_clusters=True and clustering.method=coordinate. At the time of publication, these methods are included in the auto-processing pipelines at Diamond Light Source that run during data collections where *xia*2.*multiplex* is running. Where practicable within the limits of Zenodo, raw data have been uploaded and are publicly available. Raw data from the bovine and human insulin in Section 3.1 (https://doi.org/10.5281/zenodo.15077303) and the collection of bovine, porcine and human insulin in Section 3.2.1 (https://doi.org/10.5281/zenodo.13890874) have been uploaded in full. Due to size constraints, only a subset of bovine (https://doi.org/10.5281/zenodo.15062310), porcine (https://doi.org/10.5281/zenodo.15062327) and human (https://doi.org/10.5281/zenodo.15062343) insulin from the data in Section 3.2.2 have been uploaded, alongside the outliers identified by *OPTICS* (https://doi.org/10.5281/zenodo.15062350). There is also a tutorial to process these data in *DIALS* (https://github.com/graeme-winter/dials_tutorials/blob/release-2024-12/ccp4-dls-2024/COWS_PIGS_PEOPLE.md). The same data have also been successfully separated using *XSCALE_ISOCLUSTER* from the *XDS* package, with an analogous tutorial available (https://wiki.uni-konstanz.de/xds/index.php/Scale_many_datasets).

## 5. Conclusions

In this work, we have described and demonstrated enhancements of the intensity-clustering algorithms available within *DIALS* and utilized by *xia*2.*multiplex*. Firstly, the calculation of pairwise correlation coefficients for intensity-based clustering should be appropriately weighted using the uncertainties determined from the integration and scaling of measured reflections. This has been shown to dramatically improve the quality of both the correlation and cosine-angle hierarchical clustering methods used within these programs and was necessary to enable correct dimensionality optimization and spatial density-based clustering. Over 200 data sets of bovine, porcine and human insulin at room temperature could be clustered into pure species using a σ-weighted calculation, whereas the existing unweighted methodology failed to resolve any species-pure clusters for all clustering methods. Furthermore, the appropriate number of dimensions for the *cosym* coordinate optimization is automatically optimized, which directly affects the cosine-angle HCA, providing separation of groups in cases where there are multiple types of systematic differences present. Specifically, cosine-angle HCA in at least three-dimensional space was able to separate data sets from crystals of human, bovine and porcine insulin, whereas the same analysis in two dimensions mixed the data sets of two insulin species together. Finally, inclusion of the *OPTICS* density-based clustering algorithm resulted in the automatic identification of discrete groups, including filtering out outliers in the data set as appropriate. While a similar result could be achieved with HCA, this could not be performed using proposed automatic thresholds, and thus required manual analysis, while the *OPTICS* analysis was fully automated. Our work demonstrates the sensitivity to small structural changes that can be achieved with clustering analysis. The differences in molecular weight vary in the range of 30–74 Da, constituting 0.5–1.3% of the mass of the insulin protein, and the resulting differences in the intensities could be detected down to a limit of around 100 common unique reflections on average between pairs of high-quality data sets. This technique therefore demonstrates clear applicability for automatically separating apo and ligand-bound crystals in room-temperature data collections.

## 6. Related literature

The following references are cited in the supporting information for this article: Andrews & Bernstein (2014 ▸). Winter (2010 ▸) and Zeldin *et al.* (2013 ▸, 2015 ▸).

## Supplementary Material

Experimental details, comparison between Ward and average linkage methods, OPTICS optimization and time profiling, including Supplementary FIgures and Tables. DOI: 10.1107/S2059798325004589/rr5252sup1.pdf

Cows, Pigs and People: Example data of cubic insulin from three different species recorded on Diamond Light Source I24.: https://doi.org/10.5281/zenodo.13890874

Multi-crystal in situ data set from VMXi at Diamond Light Source: bovine and human.: https://doi.org/10.5281/zenodo.15077303

Multi-crystal in situ data set from VMXi at Diamond Light Source: bovine insulin.: https://doi.org/10.5281/zenodo.15062310

Multi-crystal in situ data set from VMXi at Diamond Light Source: porcine insulin.: https://doi.org/10.5281/zenodo.15062327

Multi-crystal in situ data set from VMXi at Diamond Light Source: human insulin.: https://doi.org/10.5281/zenodo.15062343

Multi-crystal in situ data set from VMXi at Diamond Light Source: unclassified.: https://doi.org/10.5281/zenodo.15062350

## Figures and Tables

**Table 1:** Comparison of clustering methods using both the Davies–Bouldin score and V-Measure methods Lower Davies–Bouldin scores correspond to denser and well separated clusters. Higher V-Measure scores correspond to more correctly labelled data points.

	Bovine versus human (Section 3.1)		Bovine versus porcine versus human (Section 3.2.1)		Bovine versus porcine versus human (Section 3.2.2)		
Correlation HCA							
Threshold	60%	70%	60%	70%	60%	70%	47%
Davies–Bouldin score	0.204	0.204	0.460	0.834	0.799	0.682	1.492
V-Measure	1.0	1.0	1.0	0.734	0.516	0.452	0.894
Cosine-angle HCA							
Threshold	60%	70%	60%	70%	60%	70%	18%
Davies–Bouldin score	0.040	0.040	0.257	0.723	0.505	0.413	0.529
V-Measure	1.0	1.0	1.0	0.734	0.539	0.562	0.938
*OPTICS*							
Davies–Bouldin score	0.040		0.257		0.783		
V-Measure	1.0		1.0		0.856		
