# Supplementary material for: Enhanced intensity-based clustering of isomorphous multi-crystal data sets in the presence of subtle variations
Source: Acta Crystallogr D Struct Biol. 2025 May 29;81(Pt 6):278–90. doi: 10.1107/S2059798325004589 (PMC12128884; doi:10.1107/S2059798325004589)
Supplement: Supplementary file 1 [file d-81-00278-sup1.pdf]

STRUCTURAL BIOLOGY

Volume 81 (2025)

Supporting information for article:

Enhanced intensity-based clustering of isomorphous multi-crystal data sets in the presence of subtle variations

Amy J. Thompson, James Beilsten-Edmands, Cicely Tam, Juan Sanchez-Weatherby, James Sandy, Halina Mikolajek, Danny Axford, Sofia Jaho, Michael A. Hough and Graeme Winter

S1. Experimental details

S1.1. Data processing details

All diffraction data (at room and cryogenic temperatures) were automatically processed using the Xia2/DIALS pipelines (Winter, 2009, Winter *et al.*, 2022). The integrated data were reduced with `xia2.multiplex` (Gildea *et al.*, 2022) using the options `clustering.output_clusters=True` and `clustering.method=coordinate` to output significant clusters from OPTICS. For all examples without σ-weighting, the additional options `weights=none` and `cc_weights=none` were used for testing and demonstration purposes. Similarly, `dimensions=2` was used to force a lower dimensionality for comparison (Figure 4c), and `hierarchical_clustering.linkage_method=average` was used to generate figures comparing the average linkage method to the default Ward linkage method. Doses were estimated using RADDOSE-3D (Zelden *et al.*, 2013).

S1.2. Separation of Bovine and Human Insulin

S1.2.1. Bovine insulin crystallisation

Bovine insulin from Sigma-Aldrich (I5500) was dissolved in 50 mM Na_2HPO_4, 10 mM EDTA pH 10.5 to final concentration of 15 mg/ml. Crystals were grown by vapour diffusion in MiTeGen In Situ-1 plates, where 30 µl of crystallisation solution (25% (v/v) ethylene glycol), was dispensed into the reservoir. Using a Mosquito SPT liquid dispenser, with humidity control settings set to 85 % humidity, drops were dispensed mixing a 1:1 ratio of crystallisation solution to protein solution. Data were collected from crystals of mean size 44 x 43 x 22 μm^3. Crystal size represents a calculated average of crystal dimensions measured using a light microscope and images captured during beamtime.

S1.2.2. Human insulin crystallisation

Human insulin from Sigma-Aldrich (91077C) was dissolved in 50 mM Na_2HPO_4, 10 mM EDTA pH 10.5 to final concentration 13.9 mg/ml. Crystals were grown by vapour diffusion in MiTeGen In Situ-1 plates, where 30 µl of crystallisation solution (25% (v/v) ethylene glycol), was dispensed into the reservoir. Using a Mosquito SPT liquid dispenser, with humidity control settings set to 85 % humidity, drops were dispensed mixing a 1:1 ratio of crystallisation solution to protein solution. Data were collected from crystals of mean size 66 x 67 x 35 μm^3. Crystal size represents a calculated average of crystal dimensions measured using a light microscope and images captured during beamtime.

S1.2.3. Data collection parameters

Data from section 3.1 were collected at the VMXi beamline (Sanchez-Weatherby *et al.*, 2019, Mikolajek *et al.*, 2023, Sandy *et al.*, 2024). A 20° wedge of each crystal was exposed for 0.0018 s per image (0.1° per image) using a 10 x 10 μm^2 beam at 16 keV and flux of 2 × 10^{12} ph s^{-1}. X-ray diffraction data were recorded using a Dectris EIGER 2X 4M at a temperature of 293 K.

S1.2.4. Unit cell clustering

To demonstrate the structural isomorphism of these insulin crystals, the unit cell clustering is given below (Figure S1). Hierarchical clustering using the single linkage method (Zeldin *et al.*, 2015) was performed on the cell vectors in the G^6 space using the NCDist (Niggli Cone distance) metric defined by Andrews and Bernstein (Andrews & Bernstein, 2014) Alongside the histograms in the main text, it shows that unit cell clustering cannot distinguish bovine and human insulin.

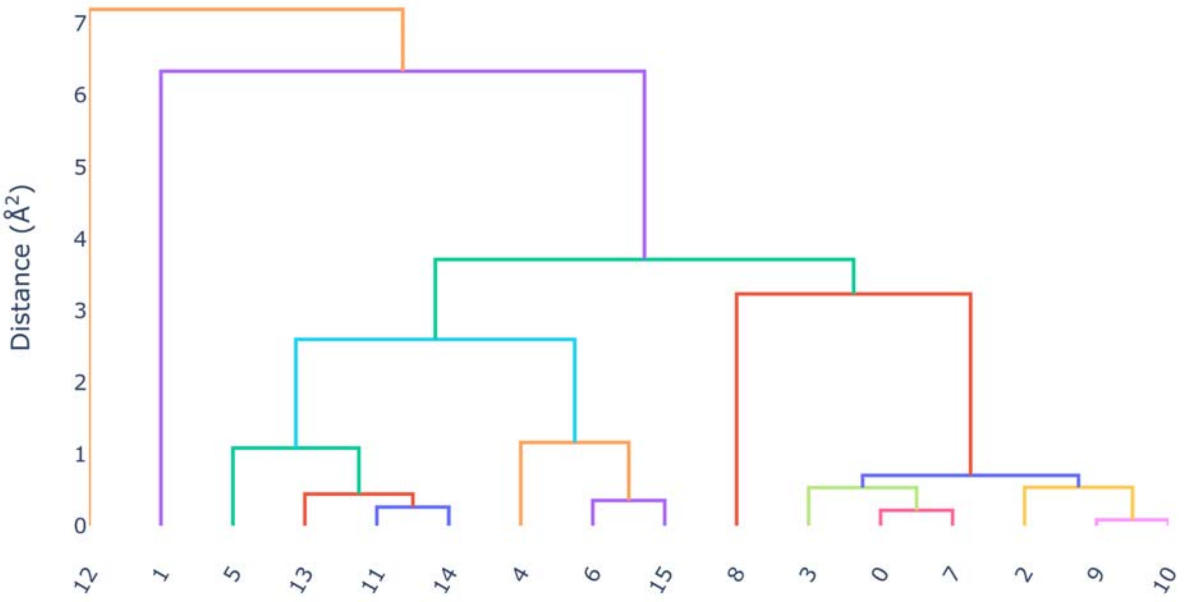

Figure S1 Unit cell clustering of human and bovine insulin. Datasets 0 – 7 correspond to bovine insulin crystals and datasets 8 – 15 correspond to human insulin crystals. There is no clear separation of the insulin species by unit cell. Dendrogram links have colours that are randomly allocated and not representative of groups.

S1.2.5. Weighted versus unweighted CC algorithm

A comparison of the correlation coefficient clustering with and without using σ-weighting in the CC calculation (Section 2.1) is given in Figure S2. Note that while the clustering is identical for all intents and purposes, the scale of the heatmap is larger when using σ-weighting, meaning the differences are more well defined.

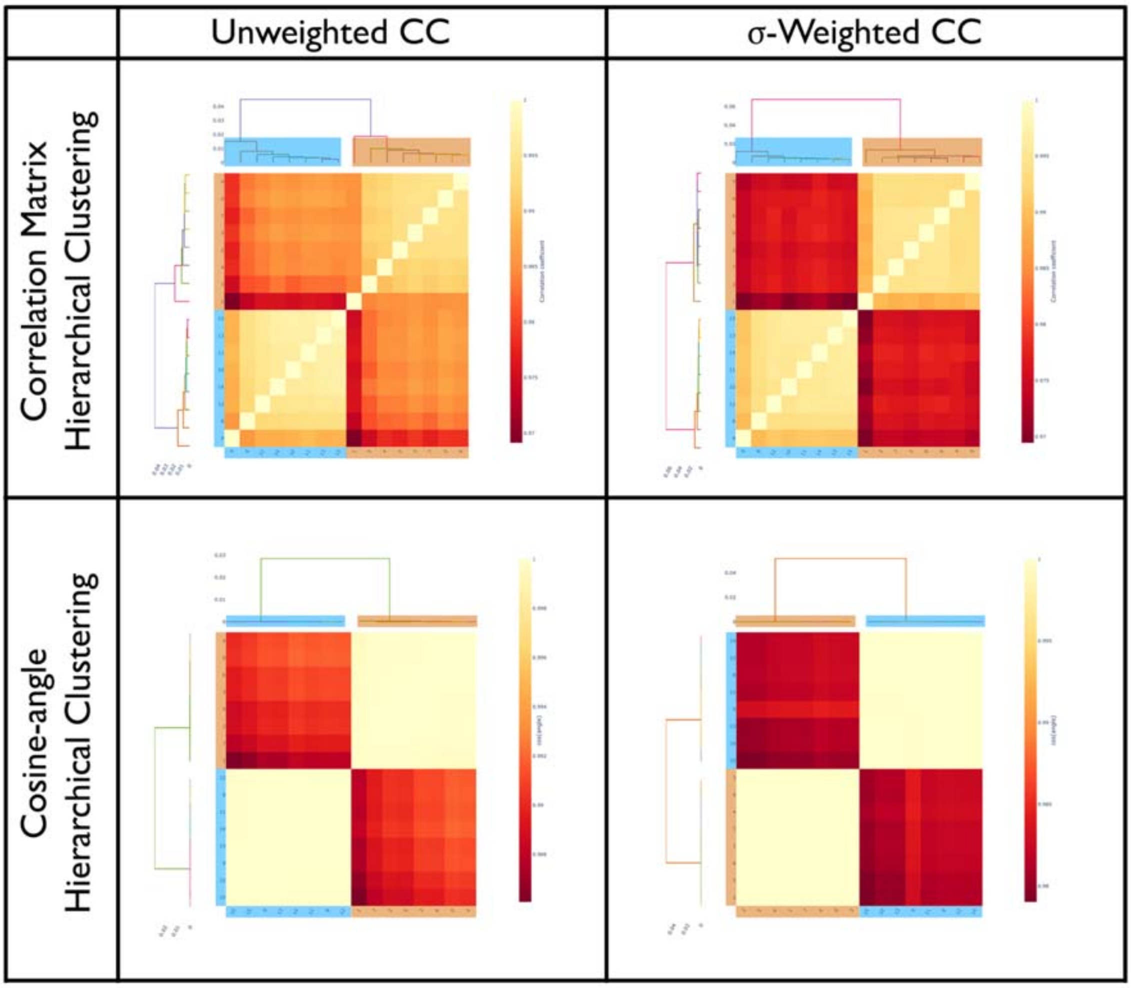

Figure S2 Comparison of the correlation-based clustering using the unweighted and σ-weighted CC calculation. Bovine insulin datasets are highlighted in orange, and human insulin datasets are highlighted in blue. Dendrogram links have colours that are randomly allocated and not representative of groups.

S1.2.6. Frequency Distributions of Correlation Coefficients

A histogram plotting the distribution of pairwise correlation coefficients is given in Figure S3. The bimodal distribution clearly shows the two species present.

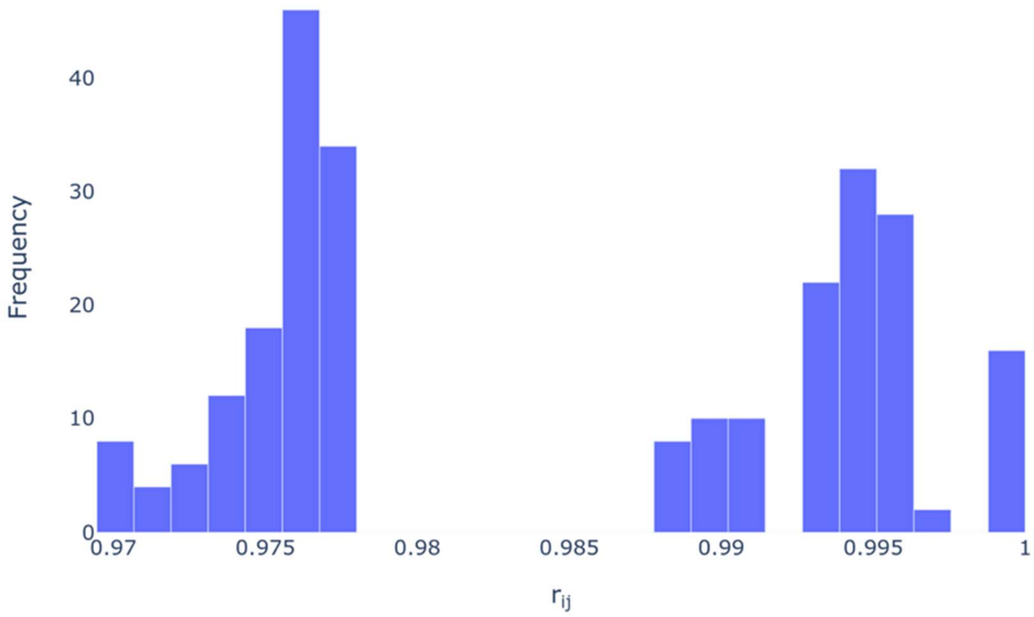

Figure S3 Histogram showing the distribution of pairwise correlation coefficients from the r_{ij} matrix (number of bins = 25).

S1.2.7. Comparison of data reduction statistics

The data reduction statistics for the entire dataset, as well as the clusters output by OPTICS are given below for comparison (Table S1). In particular, note the increase in the signal-to-noise ratio (I/σ (I)) when the clusters are separated.

Table S1 Automatic data reduction statistics for separation of bovine and human insulin at room temperature.

	**All Datasets**	**Human Cluster**	**Bovine Cluster**
Number of Datasets	16	8	8
Unit Cell (Å) (°)	78.45 78.45 78.45 90 90 90	78.44 78.44 78.44 90 90 90	78.47 78.47 78.47 90 90 90
Resolution (Å)	55.47-1.66 (1.69-1.66)	55.46-1.66 (1.69-1.66)	55.49-1.72 (1.75-1.72)
Observations	284178 (5433)	142083 (2695)	136253 (3033)
Unique Reflections	9554 (460)	9445 (447)	8471 (388)
Multiplicity	29.7 (11.8)	15.0 (6.0)	16.1 (7.8)
Completeness	98.88 % (96.03 %)	97.75 % (93.32 %)	97.27 % (91.51 %)
Mean I/σ (I)	15.6 (0.5)	21.6 (0.7)	17.6 (0.7)
R_{pim}	0.022 (0.781)	0.022 (0.869)	0.027 (0.751)
$CC_{1/2}$	0.999 (0.316)	1.000 (0.376)	0.999 (0.315)
Dose Per Crystal (kGy)	400	400	400

S1.3. Separation of bovine, porcine and human insulin – cryogenic data collection

S1.3.1. Bovine insulin crystallisation

Bovine insulin from Sigma-Aldrich (I5500) was dissolved in 50 mM Na_2HPO_4, 10 mM EDTA pH 10.5 to final concentration 23.3 mg/ml. Crystals were grown by vapour diffusion in 3 drop SwissSci plates, where 30 µl of crystallisation solution (25% (v/v) ethylene glycol) was dispensed into the reservoir. Using a Mosquito SPT liquid dispenser, with humidity control settings set to 85 % humidity, drops were dispensed mixing a 1:1 ratio of crystallisation solution to protein solution. Data were collected from crystals of mean size 70 x 65 x 32 µm^3. Crystal size represents a calculated average of crystal dimensions measured using a light microscope and images captured during beamtime.

S1.3.2. Human insulin crystallisation

Human insulin from Sigma-Aldrich (91077C) was dissolved in 50 mM Na_2HPO_4, 10 mM EDTA pH 10.5 to final concentration 24 mg/ml. Crystals were grown by vapour diffusion in 3 drop SwissSci plates, where 30 µl of crystallisation solution (25% (v/v) ethylene glycol) was dispensed into the reservoir. Using a Mosquito SPT liquid dispenser, with humidity control settings set to 85% humidity,

drops were dispensed mixing a 1:1 ratio of crystallisation solution to protein solution. Data were collected from crystals of mean size 42 x 42 x 20 μm^3. Crystal size represents a calculated average of crystal dimensions measured using a light microscope and images captured during beamtime.

S1.3.3. Porcine insulin crystallisation

Porcine insulin from Sigma-Aldrich (I5523) was dissolved in 50 mM Na_2HPO_4, 10 mM EDTA pH 10.5 to final concentration 24 mg/ml. Crystals were grown by vapour diffusion in 3 drop SwissSci plates, where 30 µl of crystallisation solution (25% (v/v) ethylene glycol) was dispensed into the reservoir. Using a Mosquito SPT liquid dispenser, with humidity control settings set to 85 % humidity, drops were dispensed mixing a 1:1 ratio of crystallisation solution to protein solution. Data were collected from crystals of mean size 36 x 38 x 30 μm^3. Crystal size represents a calculated average of crystal dimensions measured using a light microscope and images captured during beamtime.

S1.3.4. Data collection parameters

Data from section 3.2.1 were collected at the I24 beamline (Diamond Light Source). A 10° wedge of each crystal was exposed for 0.01 s per image (0.1° rotation per image) using a 30 x 30 μm^2 beam at 12.4 keV and flux of 4.5×10^{11} ph s^{-1}. X-ray diffraction data were recorded from crystals at 100 K using a Pilatus3 6M detector.

S1.3.5. Unit cell clustering

To demonstrate the structural isomorphism of these insulin crystals, the unit cell clustering is given below (Figure S4). Hierarchical clustering using the single linkage method (Zeldin *et al.*, 2015) was performed on the cell vectors in the G^6 space using the NCDist (Niggli Cone distance) metric defined by Andrews and Bernstein (Andrews & Bernstein, 2014). Alongside the histograms in the main text, it shows that unit cell clustering cannot distinguish bovine, porcine and human insulin.

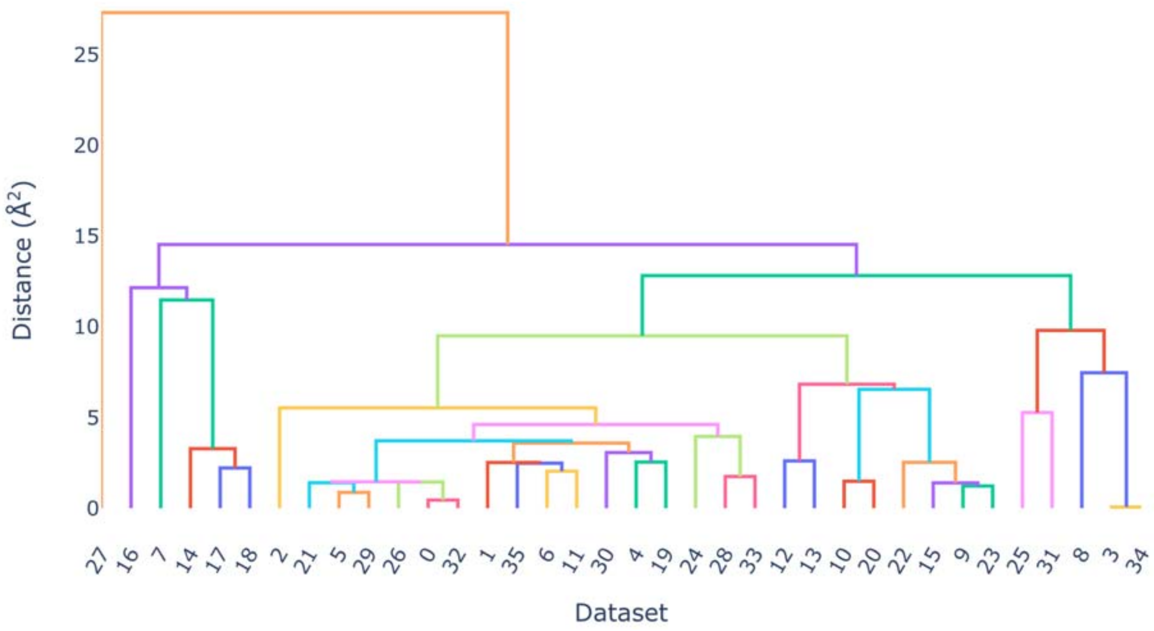

Figure S4 Unit cell clustering of human, porcine and bovine insulin. Datasets 0 – 11 correspond to bovine insulin crystals, datasets 12 – 23 correspond to porcine insulin crystals and datasets 24 – 35 correspond to human insulin crystals. There is no clear separation of the insulin species by unit cell. Dendrogram links have colours that are randomly allocated and not representative of groups.

S1.3.6. Weighted versus unweighted CC algorithm

A comparison of the correlation coefficient clustering with and without using σ-weighting in the CC calculation (Section 2.1) is given in Figure S5. Note that while the clustering is identical for all intents and purposes, the scale of the heatmap is larger when using σ-weighting, meaning the differences are more well defined.

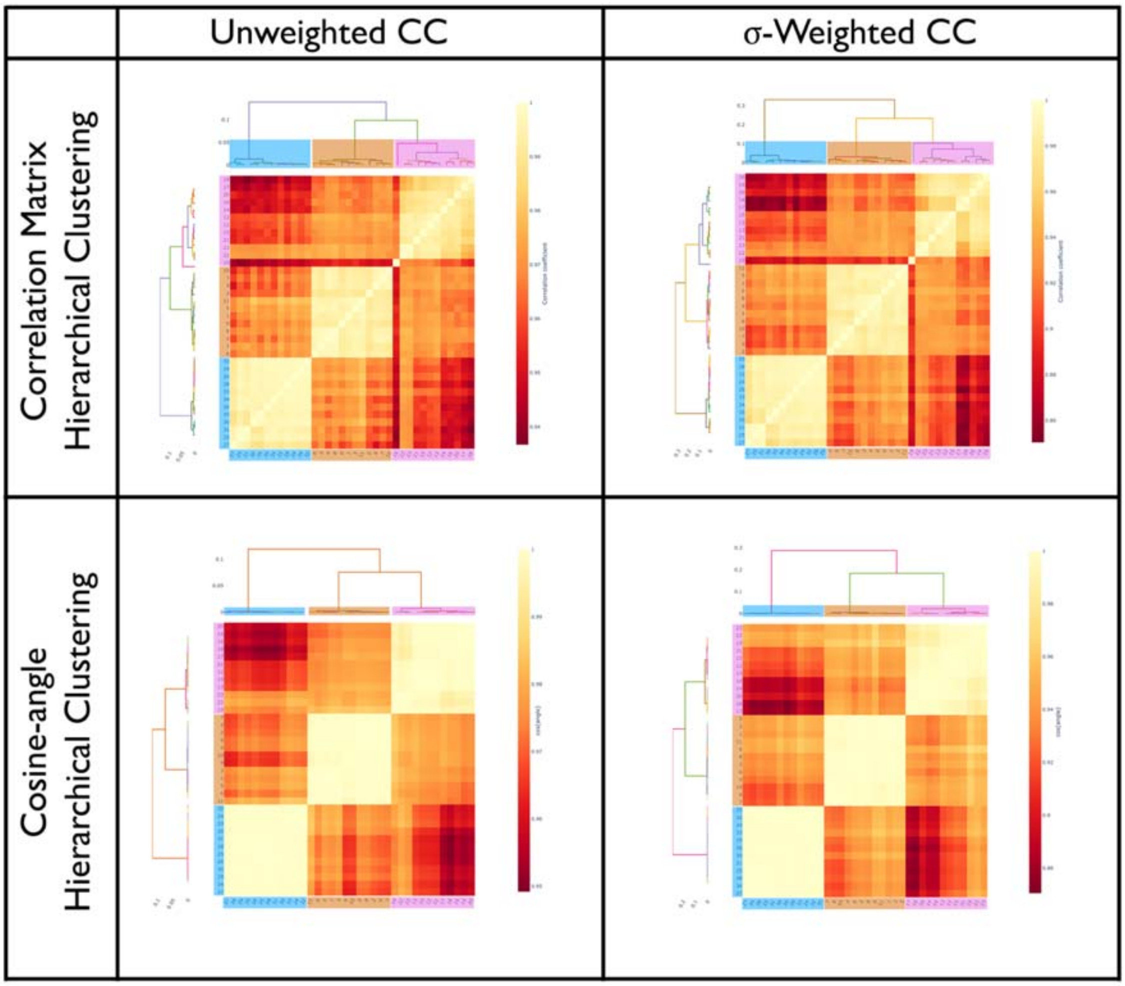

Figure S5 Comparison of the correlation-based clustering using the unweighted and σ-weighted CC calculation. Bovine insulin datasets are highlighted in orange, porcine insulin datasets are highlighted in pink, and human insulin datasets are highlighted in blue. Dendrogram links have colours that are randomly allocated and not representative of groups.

S1.3.7. Frequency distributions of correlation coefficients

A histogram plotting the distribution of pairwise correlation coefficients is given in Figure S6. There is a wide distribution of the peak centred around 0.93 as well as a sharp peak at 0.99, which is indicative of multiple species present.

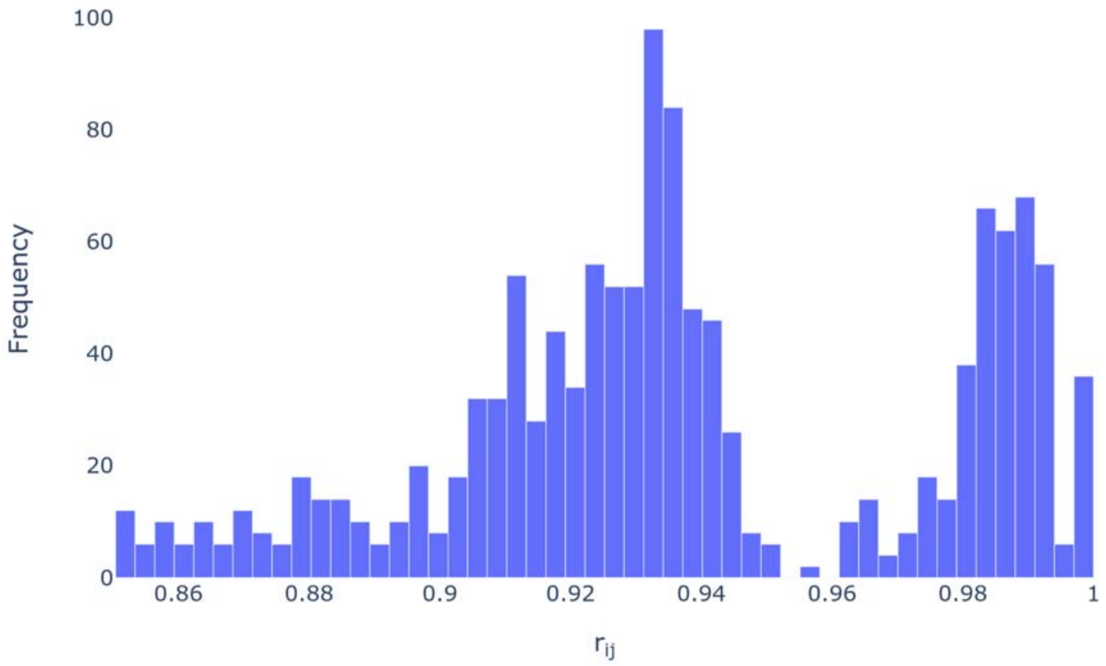

Figure S6 Histogram showing the distribution of pairwise correlation coefficients from the r_{ij} matrix (number of bins = 50).

S1.3.8. Multi-dimensional clustering graphs

When higher-dimensional analysis is required, a series of 2-dimensional projections of the optimised cosym coordinates are required to provide a comprehensive picture of the spatial data. The number of projections is dependent on the number of dimensions, with a projection provided for each unique combination of axes. The data were rotated to align with the eigenvectors identified using principal component analysis. This dataset was identified to need 3-dimensions to best represent the features in the r_{ij} matrix, and thus produces 3 orthogonal projections (Figure S7).

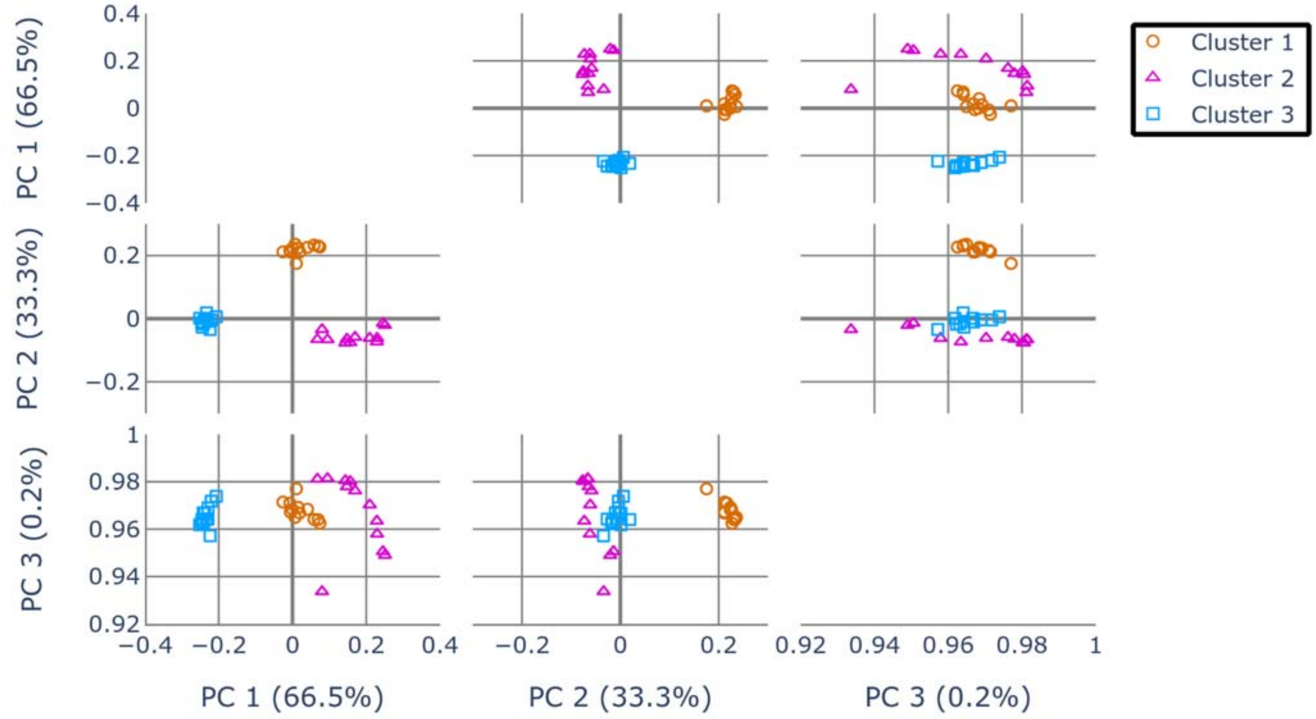

Figure S7 All 2-dimensional projections of the 3-dimensional optimised cosym coordinates, aligned with the principal components identified by PCA analysis. Clusters are labelled as identified by the OPTICS algorithm.

S1.3.9. Comparison of data reduction statistics

The data reduction statistics for the entire dataset, as well as the clusters output by OPTICS are given below for comparison (Table S2). In particular, note the increase in the signal-to-noise ratio (I/σ (I)) when the clusters are separated.

Table S2 Automatic data reduction statistics for separation of bovine, porcine and human insulin at cryogenic temperature.

	**All Datasets**	**Bovine Cluster**	**Porcine Cluster**	**Human Cluster**
Number of Datasets	36	12	12	12
Unit Cell (Å) (°)	77.89 77.89 77.89 90 90 90	77.84 77.84 77.84 90 90 90	77.69 77.69 77.69 90 90 90	78.02 78.02 78.02 90 90 90
Resolution (Å)	55.08-1.19 (1.21-1.19)	55.04-1.27 (1.29-1.27)	54.94-1.34 (1.36-1.34)	55.17-1.17 (1.19-1.17)
Observations	922728 (26351)	269845 (11703)	230249 (11006)	318385 (7604)
Unique Reflections	25338 (1266)	20843 (1028)	17656 (844)	26777 (1330)
Multiplicity	36.4 (20.8)	12.9 (11.4)	13.0 (13.0)	11.9 (5.7)
Completeness	100% (100%)	100% (100%)	99.99% (100%)	99.99% (100%)
Mean I/σ (I)	7.5 (0.2)	12.8 (0.5)	9.0 (0.4)	14.4 (0.4)
R_{pim}	0.031 (1.516)	0.024 (0.698)	0.037 (0.710)	0.018 (0.763)
$CC_{1/2}$	0.998 (0.356)	0.999 (0.333)	0.998 (0.387)	0.999 (0.320)
Dose Per Crystal (kGy)	54.9	54.9	54.9	54.9

S1.4. Separation of bovine, porcine and human insulin – room temperature data collection

S1.4.1. Bovine insulin crystallisation

Bovine insulin from Sigma-Aldrich (I5500) was dissolved in 50 mM Na_2HPO_4, 10 mM EDTA pH 10.5 to final concentration 42.1 mg/ml. Crystals were grown by vapour diffusion in MiTeGen In Situ-1 plates, where 30 µl of crystallisation solution (25% (v/v) ethylene glycol), was dispensed into the reservoir. Using a Mosquito SPT liquid dispenser, with humidity control settings set to 85 % humidity, drops were dispensed mixing a 1:1 ratio of crystallisation solution to protein solution. Data were collected from crystals of mean size 35 x 35 x 20 µm^3. Crystal size represents a calculated average of crystal dimensions measured using a light microscope and images captured during beamtime.

S1.4.2. Human insulin crystallisation

Human insulin from Sigma-Aldrich (91077C) was dissolved in 50 mM Na_2HPO_4, 10 mM EDTA pH 10.5 to final concentration 38.7 mg/ml. Crystals were grown by vapour diffusion in MiTeGen In Situ-1 plates, where 30 µl of crystallisation solution, (25% (v/v) ethylene glycol), was dispensed into the

reservoir. Using a Mosquito SPT liquid dispenser, with humidity control settings set to 85 % humidity, drops were dispensed mixing a 1:1 ratio of crystallisation solution to protein solution. Data were collected from crystals of mean size 26 x 27 x 15 µm^3. Crystal size represents a calculated average of crystal dimensions measured using a light microscope and images captured during beamtime.

S1.4.3. Porcine insulin crystallisation

Porcine insulin from Sigma-Aldrich (I5523) was dissolved in 50 mM Na_2HPO_4, 10 mM EDTA pH 10.5 to final concentration 50.5 mg/ml. Crystals were grown by vapour diffusion in MitTeGen In Situ-1 plates, where 30 µl of crystallisation solution, (25% (v/v) ethylene glycol), was dispensed into the reservoir. Using a Mosquito SPT liquid dispenser, with humidity control settings set to 85 % humidty, drops were dispensed mixing a 1:1 ratio of crystallisation solution to protein solution. Data were collected from crystals of mean size 40 x 37 x 21 µm^3. Crystal size represents a calculated average of crystal dimensions measured using a light microscope and images captured during beamtime.

S1.4.4. Data collection parameters

Data from section 3.2.2 were collected at the VMXi beamline (Sanchez-Weatherby *et al.*, 2019, Mikolajek *et al.*, 2023, Sandy *et al.*, 2024). A 60° wedge of each crystal was exposed for 0.0018 s per image (0.1° rotation per image) using a 10 x 10 µm^2 beam at 16 keV and flux of 1×10^{12} ph s^{-1}. X-ray diffraction data were recorded at 293 K using a Dectris EIGER 2X 4M detector .

S1.4.5. Unit cell clustering

To demonstrate the structural isomorphism of these insulin crystals, the unit cell clustering is given below (Figure S8). Hierarchical clustering using the single linkage method (Zeldin *et al.*, 2015) was performed on the cell vectors in the G^6 space using the NCDist (Niggli Cone distance) metric defined by Andrews and Bernstein (Andrews & Bernstein, 2014). Alongside the histograms in the main text, it shows that unit cell clustering cannot distinguish bovine, porcine and human insulin.

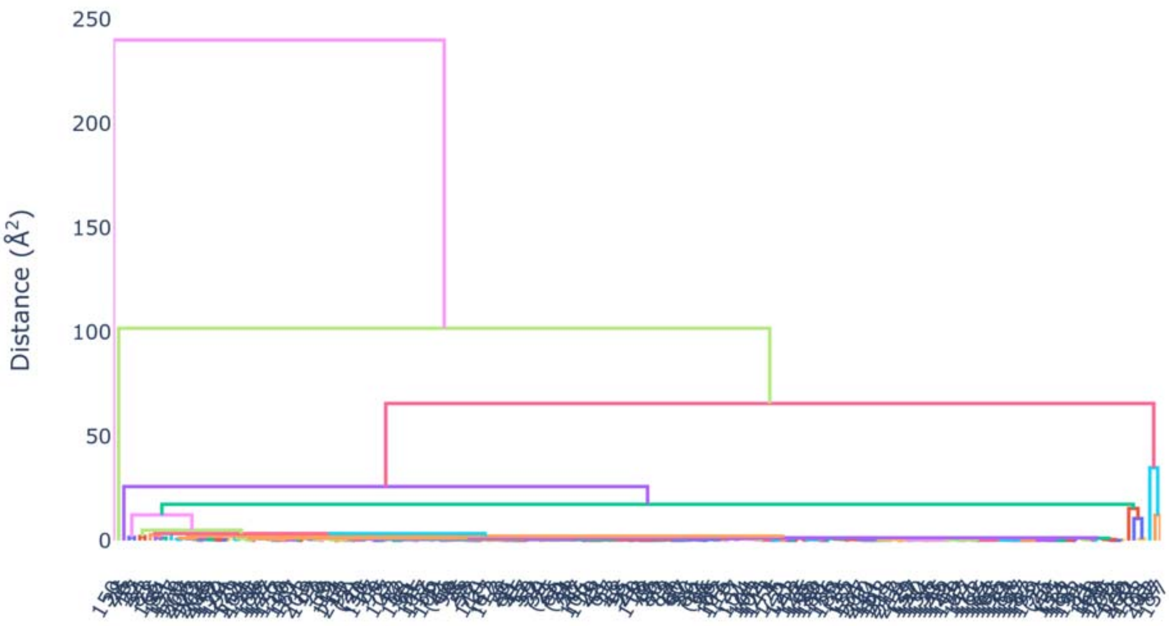

Figure S8 Unit cell clustering of human, porcine and bovine insulin. There is no clear separation of the insulin species by unit cell. Instead, occasional outliers dominate the hierarchy. Dendrogram links have colours that are randomly allocated and not representative of groups.

S1.4.6. Frequency distributions of correlation coefficients

A histogram plotting the distribution of pairwise correlation coefficients is given in Figure S9. Three clear peaks are present, indicating multiple species, as well as a broader tail indicative of the outliers present in the data.

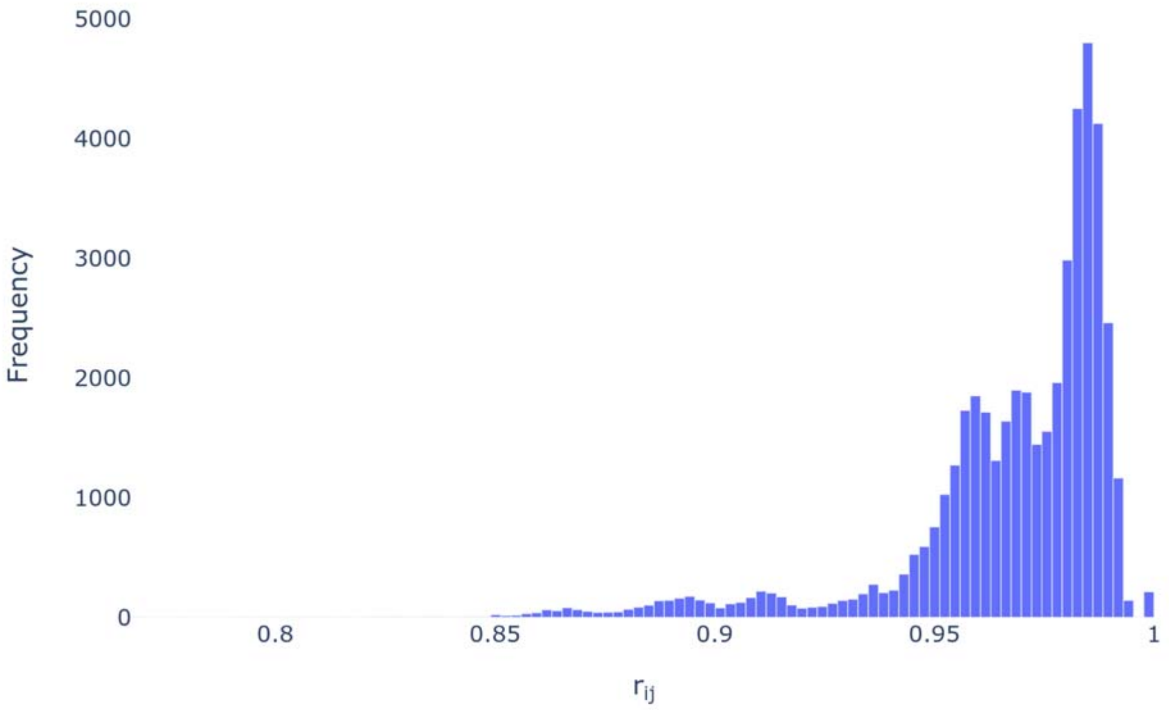

Figure S9 Histogram showing the distribution of pairwise correlation coefficients from the r_{ij} matrix (number of bins = 100).

S1.4.7. Multi-dimensional clustering graphs

When higher-dimensional analysis is required, a series of 2-dimensional projections of the optimised cosym coordinates are required to provide a comprehensive picture of the spatial data. The number of projections is dependent on the number of dimensions, with a projection provided for each unique combination of axes. The data were rotated to align with the eigenvectors identified using principal component analysis. This dataset was identified to need 4-dimensions to best represent the features in the r_{ij} matrix, and thus produces 6 orthogonal projections (Figure S10).

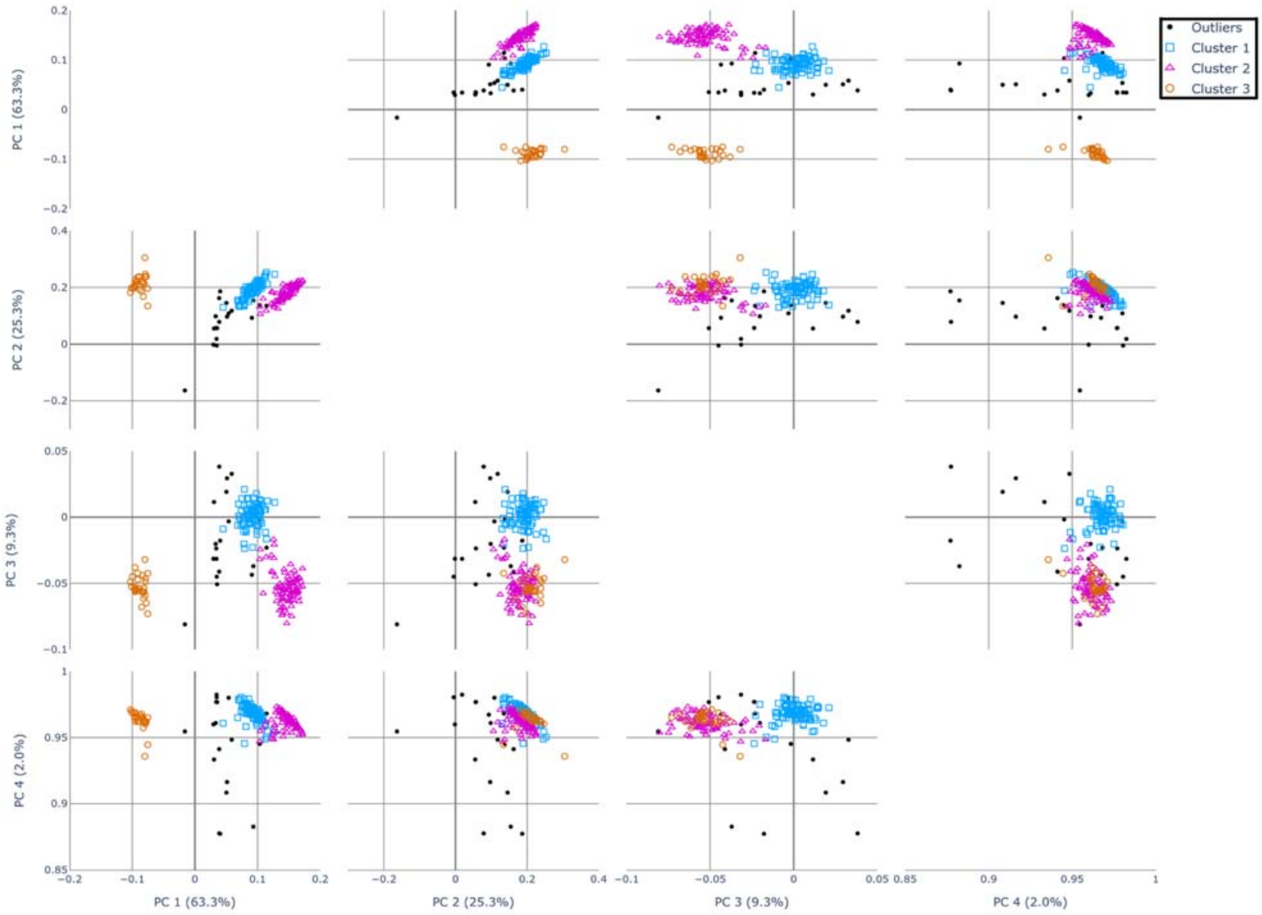

Figure S10 All 2-dimensional projections of the 4-dimensional optimised cosym coordinates, aligned with the principal components identified by PCA analysis. Clusters are labelled as identified by the OPTICS algorithm.

S1.4.8. Comparison of data reduction statistics

The data reduction statistics for the entire dataset, as well as the clusters output by OPTICS are given below for comparison (Table S3). In particular, notice the increase in overall $CC_{1/2}$ once the clusters are separated.

Table S3 Automatic data reduction statistics for separation of bovine, porcine and human insulin at room temperature.

	**All Datasets**	**Bovine Cluster**	**Porcine Cluster**	**Human Cluster**
Number of Datasets	215	28	85	83
Unit Cell (Å) (°)	78.40 78.40 78.40 90 90 90	78.42 78.42 78.42 90 90 90	78.34 78.34 78.34 90 90 90	78.42 78.42 78.42 90 90 90
Resolution (Å)	55.43-1.56 (1.59-1.56)	55.45-1.68 (1.71-1.68)	55.40-1.58 (1.61-1.58)	55.45-1.62 (1.65-1.62)
Observations	9744069 (113372)	1287298 (27519)	3697670 (49325)	3909754 (64231)
Unique Reflections	11581 (591)	9320 (474)	11141 (570)	10380 (526)
Multiplicity	841.4 (191.8)	138.1 (58.1)	331.9 (86.5)	376.7 (122.1)
Completeness	100 % (100 %)	100 % (100 %)	100 % (100 %)	100 % (100 %)
Mean I/σ (I)	38.3 (0.1)	26.1 (0.5)	38.1 (0.4)	30.6 (0.5)
R_{pim}	0.110 (-0.341)	0.142 (-1.906)	0.107 (-1.338)	0.193 (-8.798)
$CC_{1/2}$	0.969 (0.387)	0.999 (0.335)	1.000 (0.277)	1.000 (0.307)
Dose Per Crystal (kGy)	516.9	516.9	516.9	516.9

S2. Comparison between Ward and Average Linkage Methods

The performance of hierarchical clustering using both Ward and average linkage methods was assessed for all three datasets explored in this study (using the σ-weighted correlation coefficients in all cases). For the small comparison of room temperature bovine and human insulin datasets (Section 3.1), the choice of linkage method did not alter the clustering behaviour, although the Ward linkage provided marginally improved separation in the correlation clustering (Figure S11). For the comparison of cryogenic bovine, porcine and human insulin datasets (Section 3.2.1), a similar effect was observed, although the outlier dataset in the correlation clustering using the average linkage is well situated within the correct group when using the Ward linkage method (Figure S12). The most pronounced difference between the two methods, however, can be seen in the large comparison of room temperature bovine, porcine and human insulin datasets from Section 3.2.2 (Figure S13). When using the average linkage, a chain affect occurs in the correlation clustering, and while clear groups of bovine, porcine, and human insulin can be identified, the overall dendrogram is difficult to interpret, and there is no global cutoff which would provide species pure groups. When using the Ward linkage,

however, the three sub-groups are much more clearly defined in the correlation clustering. Minor improvements are also seen in the cosine-angle HCA. Based on the superior performance of the Ward linkage, this has now been made the default method in DIALS for intensity-based HCA.

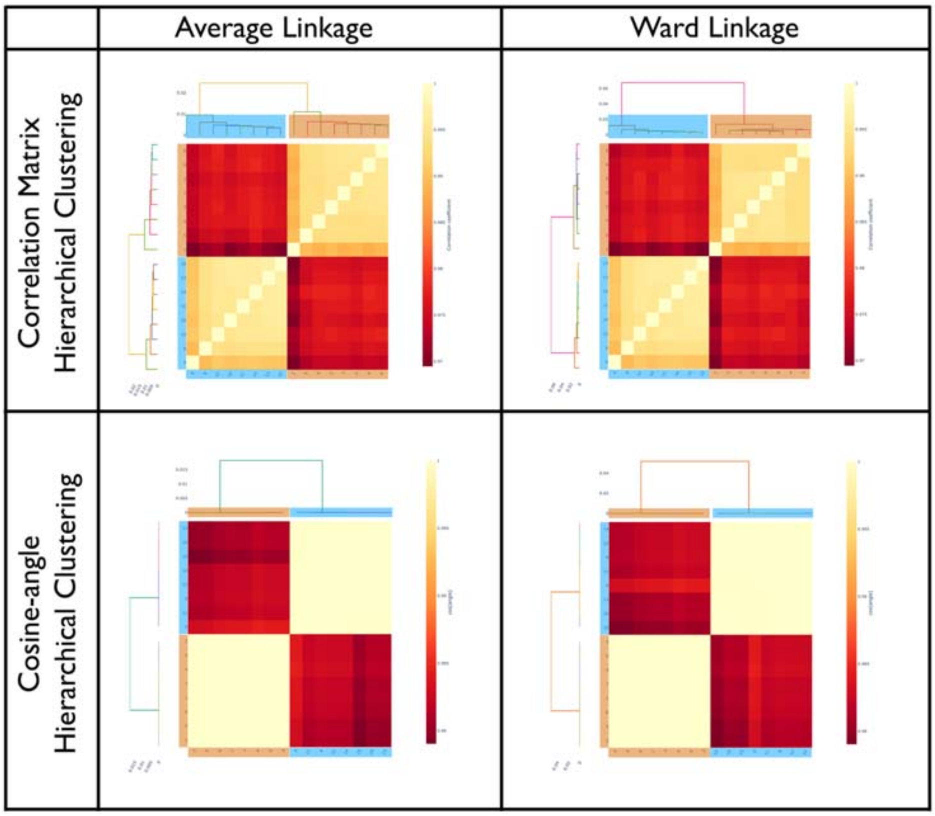

Figure S11 Comparison of the correlation-based clustering using average and Ward linkages when performing the HCA on the room temperature dataset comparing bovine insulin (orange) and human insulin (blue). Dendrogram links have colours that are randomly allocated and not representative of groups.

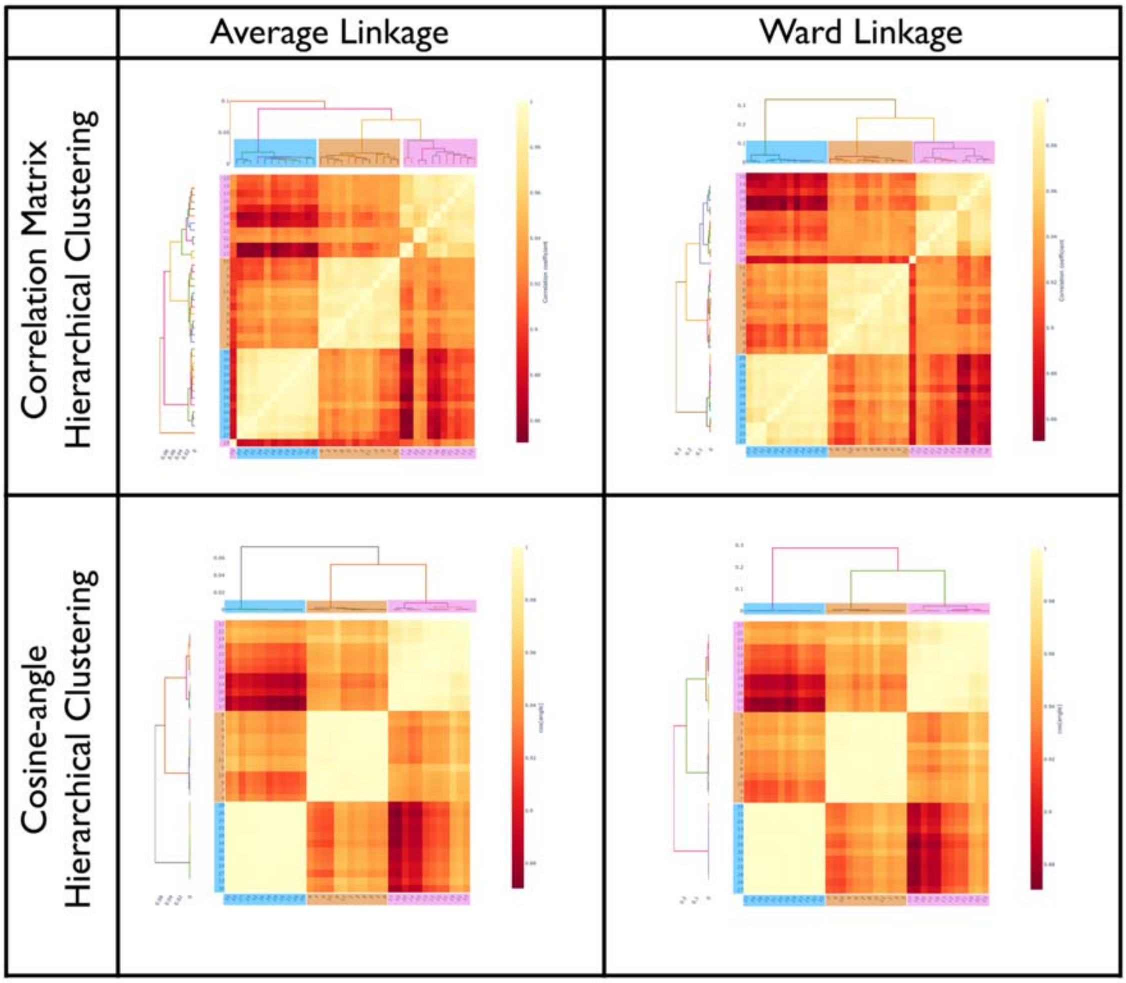

Figure S12 Comparison of the correlation-based clustering using average and Ward linkages when performing the HCA on the cryogenic dataset comparing bovine insulin (orange), porcine insulin (pink) and human insulin (blue). Dendrogram links have colours that are randomly allocated and not representative of groups.

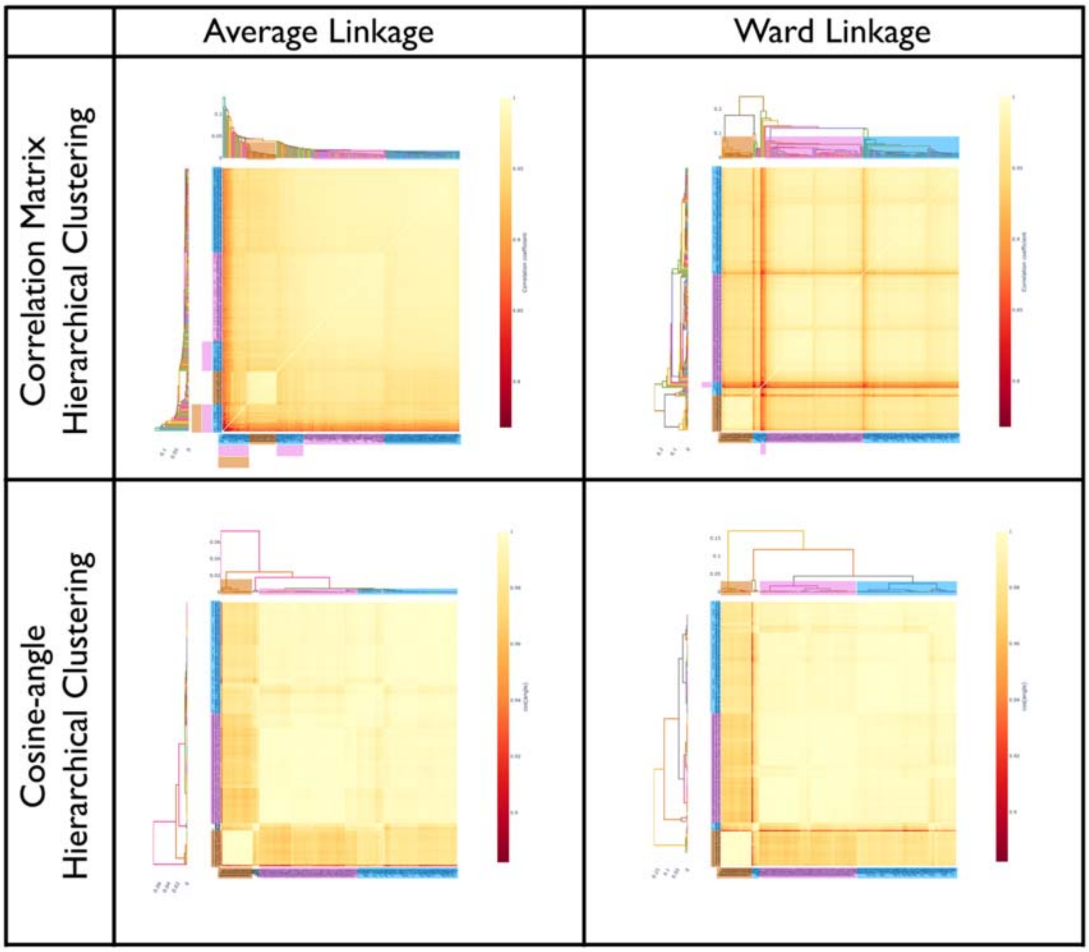

Figure S13 Comparison of the correlation-based clustering using average and Ward linkages when performing the HCA on the room temperature dataset comparing bovine insulin (orange), porcine insulin (pink) and human insulin (blue). Dendrogram links have colours that are randomly allocated and not representative of groups.

S3. OPTICS optimisation

There are currently two parameters available for optimisation through the DIALS implementation of the OPTICS algorithm: b (default 0.5) and ξ (default 0.05). While the default parameters provide appropriate clustering in the examples in this study, they may not be appropriate for all use cases. Therefore, to assist users of the software, the effect of these parameters is visually demonstrated here. A range of different outputs are demonstrated using the large room temperature separation of bovine, porcine and human insulin (Figure S14 – S17). For this dataset, the clustering is successful when b is between 0.52 and 0.45 for default ξ. The clustering is also successful when ξ is between 0.178 and 0 for default b.

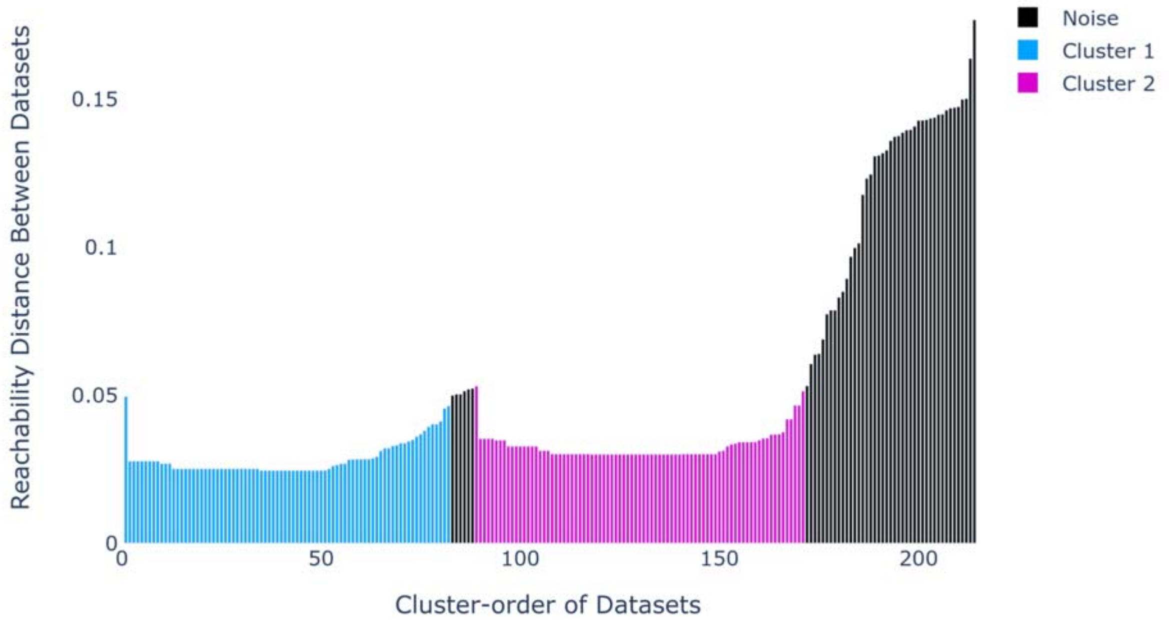

Figure S14 Reachability plot for the large room temperature separation of bovine, porcine and human insulin for b = 0.9 and ξ = 0.05. When b is far too large, the features of the reachability plot are smoothed out and smaller clusters may no longer be identified.

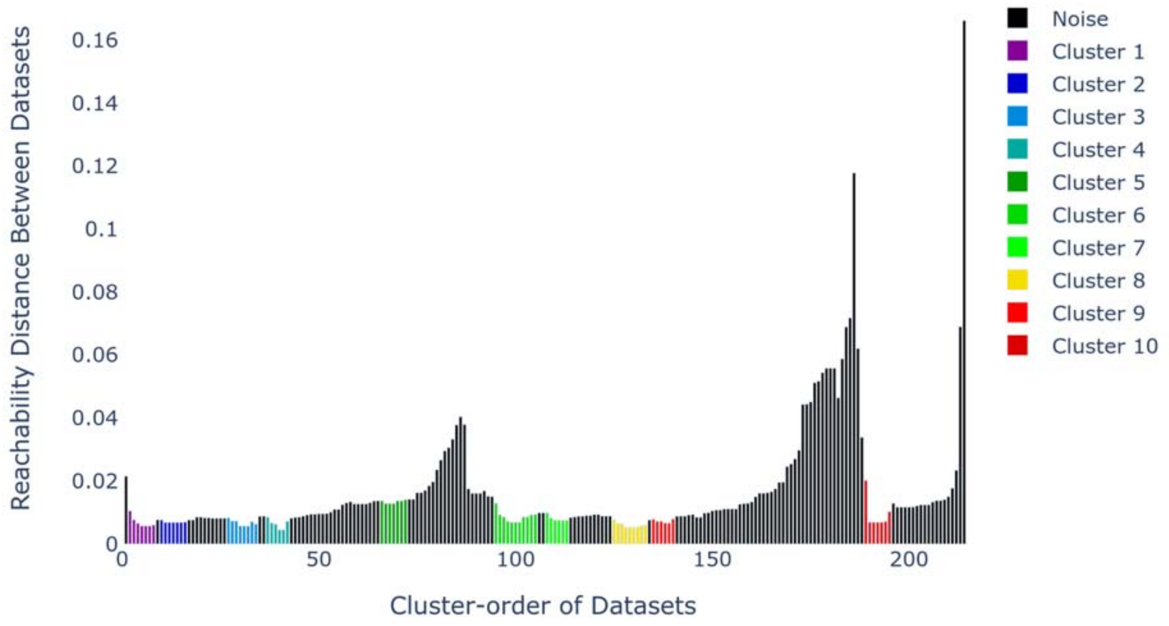

Figure S15 Reachability plot for the large room temperature separation of bovine, porcine and human insulin for b = 0.05 and ξ = 0.05. When b is far too small, the reachability plot is far coarser, resulting in many very small clusters rather than larger ones.

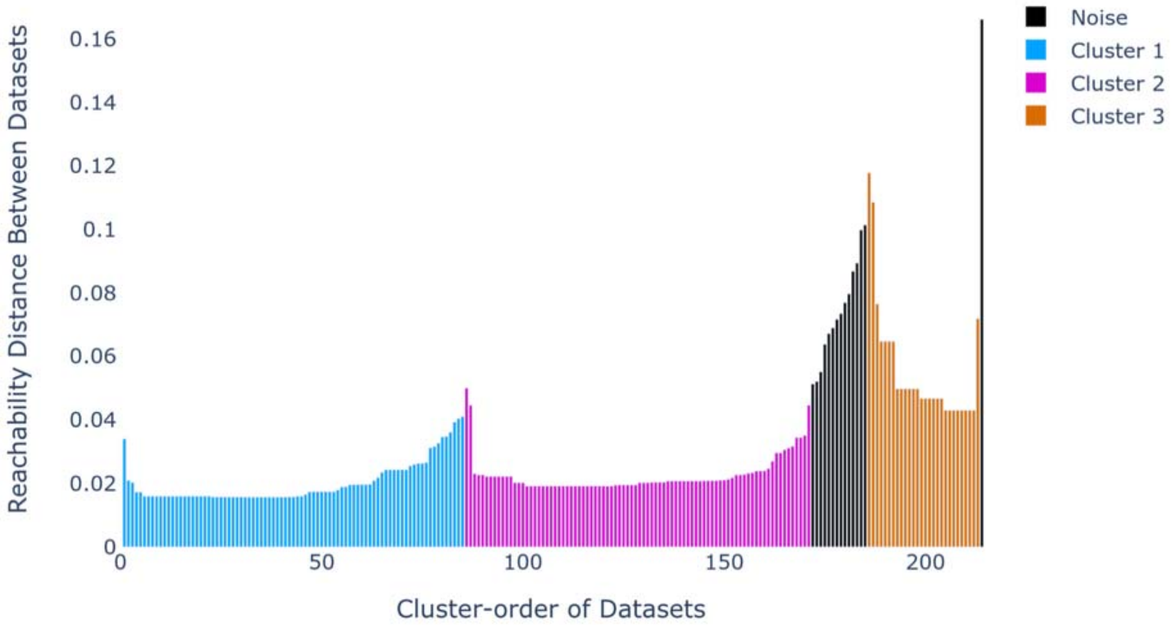

Figure S16 Reachability plot for the large room temperature separation of bovine, porcine and human insulin for b = 0.5 and ξ = 0. In this case, clustering is still successful even with the minimum value of ξ, although fewer outliers are identified, thus the resulting datasets may be noisier.

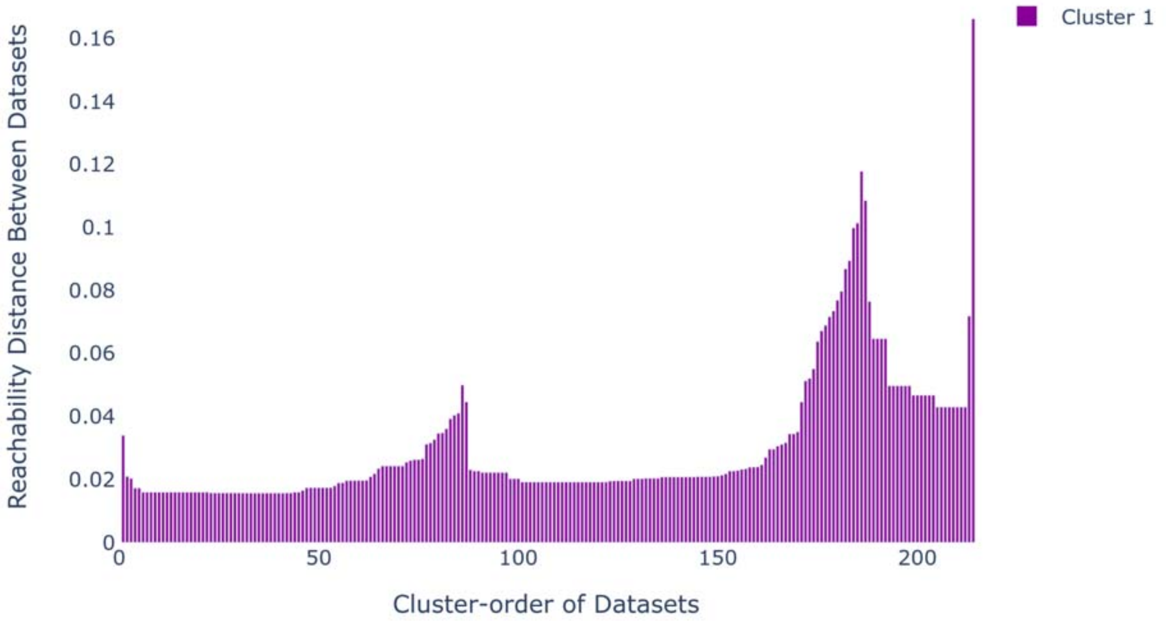

Figure S17 Reachability plot for the large room temperature separation of bovine, porcine and human insulin for b = 0.5 and ξ = 0.3. When ξ is far too large, features in the reachability plot are not accounted for and all the data is classified as a single group.

S4. Time profiling

The large room temperature comparison of bovine, porcine and human insulin was cut down to 16, 32, 64 and 128 datasets to compare computation times of key steps in the clustering analysis (performed using `dials.correlation_matrix`). The results are presented in Table S4, where all jobs were run on a computing cluster node using 16 AMD EPYC7302 CPUs and 2GB memory resource per CPU. Note that the calculation of the r_{ij} matrix is the only stage that utilises multiprocessing.

Table S4 Time profiling results to demonstrate computational requirements of key stages of clustering in DIALS. Note that the calculation of the cosine coordinates is equivalent to a single execution of the dimension optimisation step (rather than testing a series of possible dimensions).

	r_{ij}	Dimension Optimisation	Cosine Coordinates	Correlation HCA	Cosine HCA	OPTICS
16 Datasets	1.186 s	0.5472 s	0.03610 s	0.0009372 s	0.0009882 s	0.1452 s
32 Datasets	2.456 s	2.1181 s	0.05227 s	0.0009639 s	0.001031 s	0.2704 s
64 Datasets	6.064 s	6.382 s	0.09398 s	0.001014 s	0.001321 s	0.5189 s
128 Datasets	21.84 s	15.95 s	0.2663 s	0.001191 s	0.003331 s	1.025 s
216 Datasets	53.24 s	31.00 s	0.4135 s	0.001523 s	0.004308 s	1.730 s
